# Modulatory Properties of Vitamin D in Type 2 Diabetic Patients: A Focus on Inflammation and Dyslipidemia

**DOI:** 10.3390/nu15214575

**Published:** 2023-10-27

**Authors:** Rizqah MacGirlley, Wendy N. Phoswa, Kabelo Mokgalaboni

**Affiliations:** Department of Life and Consumer Sciences, College of Agriculture and Environmental Sciences, University of South Africa, Florida Campus, Private Bag X6, Roodepoort 1710, South Africa; 10916970@mylife.unisa.ac.za (R.M.); phoswwn@unisa.ac.za (W.N.P.)

**Keywords:** vitamin D, inflammation, dyslipidemia, type 2 diabetes

## Abstract

Background: Evidence from preclinical studies has found a correlation between the development of type 2 diabetes (T2D) and vitamin D deficiency. However, evidence from randomized controlled trials (RCTs) revealed inconclusive results on vitamin D supplementation. We explored the effect of vitamin D on inflammation and dyslipidemia in T2D. Methods: We comprehensively searched for RCTs evaluating the effect of vitamin D in T2D on PubMed. Data were analyzed using Review Manager 5.3 and reports, such as standardized mean difference (SMD) and 95% confidence intervals (CI) at a 5% significant level using a random effect model. Results: This study revealed a significant reduction in tumor necrosis factor-alpha (TNF-α) SMD = (−0.51, 95%CI (−0.93, −0.09); *p* = 0.02), high sensitivity C-reactive protein (hs-CRP) SMD = (−1.06, 95%CI (−1.67, −0.45); *p* < 0.05) in vitamin D compared to placebo. Additionally, interleukin-6 (IL-6) exhibited a marginal effect SMD = (−0.52, 95%CI (−1.05, 0.01), *p* = 0.05). Furthermore, a significant reduction in the level of triglycerides SMD = (−0.65, 95%CI (−1.11, −0.18), *p* < 0.05) was observed, concomitant to a significantly increased high-density lipoprotein (HDL) level SMD = (0.53, 95%CI (0.08, 0.98), *p* = 0.02). However, no statistically significant changes were observed in total cholesterols SMD = (−0.16, 95%CI (−0.57, 0.24), *p* = 0.43) and low-density lipoprotein (LDL) SMD = (−0.06, 95%CI (−0.37, 0.24), *p* = 0.67). Conclusions: These findings suggest that vitamin D supplementation may be beneficial in ameliorating inflammation and dyslipidemia in T2D patients.

## 1. Introduction

Type 2 diabetes mellitus (T2D) is a chronic disease associated with an increased mortality rate [1] due to impaired pathways that regulate homeostatic and inflammatory responses. A recent report by the International Diabetes Federation (IDF) indicates that 537 million people are estimated to have diabetes worldwide, which is anticipated to rise to 783 million by 2045 [2]. This is alarming given the association between T2D and the risk of cardiovascular diseases (CVD), resulting in a financial burden on the healthcare system [3,4].

Although glucose-lowering pharmacological agents [5] are widely used in T2D, the death rate is continuously increasing due to secondary complications associated with diabetes [5]. Moreover, the T2D population reportedly dies from CVD more frequently than healthy individuals [6]. T2D is associated with obesity, and a correlation exists among body weight, insulin resistance, dyslipidemia, and hypertension [6,7,8]. Hyperglycemia and persistent inflammation in T2D contribute to vascular damage [9,10,11]. This further promotes the progression of CVD. Other studies have shown an increased risk of CVD and secondary complications amongst T2D patients who rely on pharmacological drugs for extended periods [12,13,14]. Thus, this is the motivation for exploring different pharmacological agents to find one with potent antihyperglycemic and hypolipidemic potential.

Other studies have explored the beneficial impact of natural antioxidants on glucose control, inflammation, and lipid metabolism in metabolic conditions [15,16,17]. This is crucial as poor glucose tolerance in T2D has been attributed to an exacerbated pro-inflammatory response and endothelial dysfunction brought on by oxidative stress. There has consistently been a significant interest in exploring the pathways and mechanisms by which dietary antioxidant compounds protect against diabetes-related complications due to their potential effects on inflammation and oxidative stress [18].

Many dietary compounds [19], including vitamin D, are widely used for distinct antioxidant potential. Vitamin D, also called calciferol [20,21], is a membrane antioxidant and a member of a fat-soluble vitamin that alleviates inflammation by inhibiting nuclear-factor-kappa-β (NF-κβ) activity [22,23]. The direct stimulation of pancreatic β-cells to release insulin [24], anti-inflammatory [25] effects to reduce chronic inflammation brought on by insulin resistance, and downregulation of elevated parathyroid hormone levels [26] that inhibit insulin secretion [27] are some of the protective mechanisms of vitamin D that have been proposed. In addition to being present in dietary supplements, a small amount can be found in oily fish, red meat, egg yolk, liver, and fortified cereals [21].

The previous meta-analysis confirmed the beneficial effects of vitamin D in regulating blood glucose; however, the improvement was minimal [28]. This effect might be attributed to vitamin D’s unique property in alleviating oxidative stress and inflammation [29]. Although two previous quantitative studies conducted in 2018 have shown that vitamin D supplementation in T2D patients can reduce inflammation [30,31] and lipid profiles [32], the results are inconsistent, and the markers evaluated were not common in both these reviews. Notably, the above studies did not focus on these parameters simultaneously to explore the effect of vitamin D on T2D. This led us to comprehensively and systematically review and meta-analyze the evidence from randomized controlled trials (RCTs) simultaneously to evaluate [32] the potential benefits of vitamin D in all these parameters. This information is important for understanding the health benefits of this dietary antioxidant in order to reduce the number of diabetic individuals who succumb to CVD complications associated with T2D. Therefore, in this study, we aimed to evaluate the overall effect of vitamin D supplementation on inflammatory markers and lipid profiles in T2D patients.

## 2. Materials and Methods

### 2.1. Study Design and Registration

This meta-analysis was conducted according to PICO guidelines [33] and reported per the Preferred Reporting Items for Systematic Reviews and Meta-Analysis (PRISMA) guidelines [34]. The protocol accompanying this review has been registered with the International Platform of Registered Systematic Review and Meta-Analysis Protocols (INPLASY), registration number (INPLASY202260022), and further published [35].

### 2.2. Adapted Search Strategy and Information Sources

Two independent researchers (RM and KM) identified the eligible studies indexed in the PubMed database using the following MesH terms and Booleans: (“vitamin D” OR “calciferol” OR “1,25-Dihydroxycholecalciferol”) AND (“Type 2 diabetes mellitus” OR “Type 2 diabetes” OR “hyperglycemia” (Supplementary Table S1). The third researcher, WNP, was invited for arbitration if RM and KM disagreed. The last updated search was conducted on 21 June 2023. References of relevant studies were screened manually to identify potentially eligible studies that might have been missed on PubMed. An exact search strategy on PubMed was updated on 21 June 2023 and attached in Supplementary Table S1.

### 2.3. Study Selection Procedure

**Figure 1:**
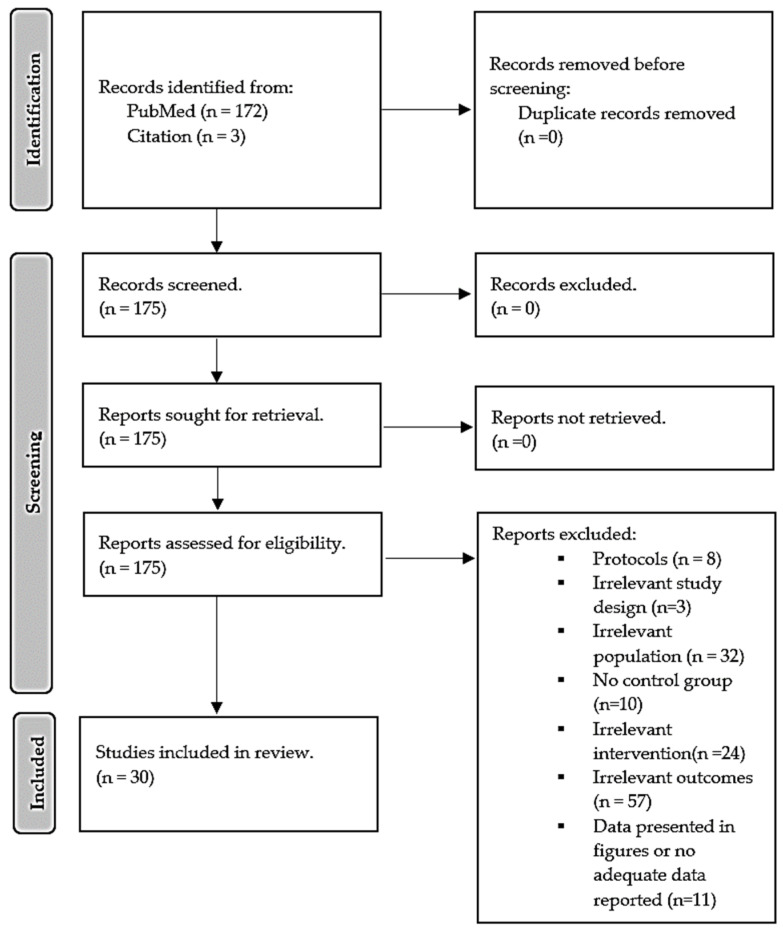
Preferred Reporting Items for Systematic Review and Meta-Analysis (PRISMA) flow diagram illustrates the trial selection procedure.

The eligibility criteria followed a PICO guideline [26]: population (P)-adult type 2 diabetes patients, Intervention (I)-vitamin D; comparator (C)-patient on placebo treatment or patient without treatment; outcomes (O)-lipid profile and inflammatory markers, study design-randomized controlled trials (RCTs). We selected the recent trial where the author conducted multiple trials using the same sample size. We excluded studies conducted in children, not on diabetes or using vitamin D supplementation in combination with other treatments, experimental models of diabetes, reviews, letters and commentaries, case-control, and cross-sectional studies. The flow diagram in Figure 1 was created following the PRISMA guide.

### 2.4. Data Extraction, Quality, and Certainty of Evidence Assessment

The following data items were extracted independently by RM and KM: first author and year of publication, the country where the study was conducted, type of study design, population size and the number of patients, description of participants, gender distribution, baseline age of recruited participants, the form of vitamin D, dose, duration of intervention, and outcome measures. Subsequently, RM and KM independently evaluated the study quality using the Jadad scale [36]. This technique considers five domains for evaluating the quality of RCTs in which three or more scores indicate excellent quality. The overall certainty of the evidence obtained from this meta-analysis was evaluated according to the Grading of Recommendations Assessment, Development, and Evaluation (GRADE) guideline [37]. This takes into account the high risk of bias, imprecision, indirectness, heterogeneity, publication bias, and effect size, and these were assessed using the GRADEprofiler tool (https://www.gradepro.org/ accessed on 14 August 2023) and RevMan software (version 5.4). Overall, evaluation results were presented as “very low”, “low”, “moderate”, or “high” risk, presented as a summary of findings in Table S4 of the Supplementary File. Any disagreements were resolved through discussion with a third independent researcher (WNP) and re-evaluating the study or domain in dispute.

### 2.5. Subgroup, Sensitivity Analysis, and Publication Bias

Subgroup analyses [38] were conducted to investigate the source of heterogeneity. Briefly, this was performed by subgrouping studies based on the dosage of vitamin D and duration of intervention, and this was classified as low- and high-dose, short- and long-term, respectively. To test the stability of the analyzed results, we conducted a leave-out one-study sensitivity analysis to recalculate the effect size of all outcomes. Lastly, for the risk of publication bias, funnel plots and the Egger regression test were used to graphically present and statistically assess the potential publication bias [39]. An Eggers test *p*-value less than 0.05 is classified as potential evidence of bias, while *p* > 0.05 signifies no bias.

### 2.6. Data Synthesis and Statistical Analysis

All extracted data were analyzed using Review Manager [Computer program], Version 5.4, The Cochrane Collaboration, 2020, and metaUN web tool (http://softmed.hacettepe.edu.tr/metaHUN/) accessed on 20 October 2023. For this meta-analysis, continuous data were extracted from RCTs as a change in mean and standard deviation (SD); however, in case a change in mean and SD was not reported, we calculated the mean difference between the baseline (pretest) and final results (post-test) (∆Mean = (M_f_ − M_b_)) and further estimated change in SD by using the formula: $\Delta SD\;=\;\sqrt{{\left({SD}_{b}\right)}^{2}+{\left({SD}_{f}\right)}^{2}-2(r\times {SD}_{b}\times {SD}_{f})}$ outlined by Cochrane guidelines and other researchers, where r, a correlation coefficient of 0.7, was used in both groups in accordance with the previous reports [40,41,42]. In cases where non-parametric data were reported (median, range, or interquartile range (IRQ), we used Hozzo et al., 2005 [43] or $SD\;=\;\frac{IQR}{1.35}$ [44] accordingly to estimate mean and SD. On the other hand, if the standard error of the mean (SEM) was reported, we estimated SD using the equation $SEM\;=\;\frac{SD}{\sqrt{n}}$ [45]. All data are presented as standardized mean differences (SMD) and 95% confidence intervals (CI), partly due to different units of measurement used in the trials. Heterogeneity between studies was determined using the *I*^2^ statistic [46] (*I*^2^ ≥ 50%) was considered significant. A random-effect model was applied for a statistically significant heterogeneity [36]. To test the stability of the analyzed results, we conducted a leave-out one-study sensitivity analysis to recalculate the effect estimates. For converting vitamin D from microgram (µg) to an International Unit (IU), we assumed that 1 IU equals 0.025 µg.

## 3. Results

### 3.1. Comprehensive Search and Information Sources

Our search from the main database, PubMed, yielded about 172 randomized controlled trials, and through hand screening of relevant references, three additional trials were further identified. Study selection was made independently by KM and RM; where there was disagreement, we sought intervention from a third researcher, WNP, for her independent opinion and further discussed with her to reach a conclusion based on the study in question. The selection procedure was a two-step process, initially screening trials through titles, abstracts, and keywords followed by a full-text screening for eligibility. From 165 records, hundred twenty-seven records were excluded based on the following reasons: (1) about 57 had no outcome of interest, (2) eight study protocols, (3) ten trials without control, (4) three irrelevant designs, (5) 32 irrelevant population (pre-diabetes, diabetic neuropathy, gestational diabetes, and chronic kidney diseases), (6) 24 different interventions (metformin, and statin), (7) eleven trials presented data in figures or did not report change after a period of intervention. Therefore, 27 trials from PubMed were found relevant in addition to 3 from the citation list. Hence, this systematic review and meta-analysis consisted of evidence from 30 trials with sufficient data pooled for meta-analysis on different effect measures. A detailed selection procedure is presented as the PRISMA flow diagram in Figure 1.

### 3.2. General Characteristics of Included Randomized Controlled Trials

The general overview of included randomized controlled trials (RCTs) is shown in Table 1. In brief, all included studies were RCTs published in peer-reviewed journals between 2010 and 2022. The participants recruited from these trials in the intervention group were 2310 who received vitamin D as treatment. Although all included trials were on T2D, one trial also considered patients who were T2D and obese [47]. Briefly, the vitamin D group patients ranged from 8 to 703 participants.

These RCTs were conducted in 14 countries, with at least ten trials conducted from Iran [48,49,50,51,52,53,54,55,56,57], three from the United States of America [58,59,60], Canada [61,62,63], and Italy [64,65,66], respectively; at least one from each of the following countries: Australia [67], China [68], Denmark [69] India [70], Israel [71], Korea [72], Mexico [73], Singapore [74], Switzerland [75], and United Arab Emirates [47]. The mean age of the included participants was 57.80 ± 7.01 years. Regarding gender distribution, 1132 (49.1%) males and 1178 females (50.9%) were on vitamin D treatment. The vitamin D was administered at different doses from as low as 10 IU (0.25 µg) to 300,000 IU (7500 µg) of vitamin D. Duration of intervention ranged from a short period (3 weeks) to a longer period (5 and half years). Different markers of inflammation and lipid profiles were determined from these studies.

**Table 1:** Characteristics of included randomized controlled trials.

Author, Year	Study Design, Country	Population and Sample Size	Intervention Group, n. Male, n (%)	Mean Age of Intervention Group (Years)	Vitamin D, Dosage, and Duration of Intervention	Effect on Lipids and Inflammatory Markers
Hu et al., 2022 [68]	Randomized controlled trial (RCT); China	T2D patients, 220	115; 32 (27.8)	66.05 ± 9.35	Oral dose of 800 IU (two capsules) of vitamin D3 for 30 months.	Vitamin D supplementation significantly decreased total cholesterol (TC) and CRP.
Hoseini et al., 2022 [57]	Randomized, single-blinded, placebo-controlled clinical trial; Iran	T2D patients, 20	10; 10 (100)	49.10 ± 1.23	50,000 IU of Vitamin D capsules per week for eight weeks.	Vitamin D supplementation significantly reduced tumor necrosis factor-alpha (TNF-α) and CRP.
Limonte et al., 2021 [58]	Randomized double-blinded placebo-controlled trial; USA	T2D patients, 1312	703; 376 (53.5)	67.4 ± 7.0	2000 IU vitamin D3 per day for five years.	Vitamin D supplementation showed no significant changes in interleukin (IL)-6 and CRP compared to the placebo group.
Barale et al., 2020 [65]	Open-label randomized-controlled pilot study; Italy	T2D patients, 30	16; 11 (68.8)	71.6 ± 3.5	500 IU oral cholecalciferol once a week for one year.	Vitamin D supplementation showed no significant difference in lipid profiles compared to the placebo group.
Hajj et al., 2020 [76]	Randomized controlled double-blind study; Lebanon	T2D patients, 88	45; 23 (51)	66.9 ± 4.1	10,000 IU cholecalciferol three times per week for six months.	Vitamin D supplementation significantly reduced CRP and TNF-α levels compared to baseline data. No significant changes were observed in terms of IL-6 when compared to baseline data.
Imanparast et al., 2020 [56]	Randomized placebo-controlled trial; Iran	T2D patients, 92	23; 11 (47.8)	53.63 ± 12.29	50,000 IU of vitamin D3 per week for four months	Vitamin D supplementation significantly decreased TC and TNF-α levels, with no significant changes in LDL, HDL, and triglyceride (TG) levels compared to the placebo group.
Meng et al., 2020 [59]	Double-blinded randomized placebo-controlled trial USA	T2D patients, 127	56; 43 (76.8)	65 ± 8.0	4000 IU per day of vitamin D3 for 24 weeks.	Vitamin D supplementation showed no significant difference in serum LDL and HDL-c compared to the placebo.
Mirzavandi et al., 2020 [55]	Randomized, controlled clinical trial; Iran	T2D patients, 50	25; 5 (20)	46 ± 1.0	Two intramuscular injections of a 200,000 IU vitamin D supplement at 0 and 4 weeks.	Vitamin D supplementation led to a significant decrease in the levels of CRP and TG in comparison to baseline data.
Dadrass et al., 2019 [54]	Randomized, placebo-controlled, double-blinded clinical trial; Iran	T2D patients, 24	12; 12 (100)	53.83 ± 6.61	50,000 IU per 2 weeks for three months	Vitamin D supplementation significantly decreased IL-6 and TNF-α without change in CRP compared to placebo.
Omidian et al., 2019 a [52]	Randomized double-blinded placebo-controlled trial; Iran	T2D patients, 66	32; 19 (59.4)	49.7 ± 6.5	4000 IU vitamin D daily for 12 weeks.	Vitamin D supplementation significantly increased TG levels compared to baseline data.
Omidian et al., 2019 b [53]	Parallel randomized double-blind placebo-controlled clin- ical trial; Iran	T2D patients, 47	24; 10 (41.6)	51.3 ± 4.7	4000 IU vitamin D daily for 12 weeks.	Vitamin D supplementation significantly decreased IL-6 and MCP-1 levels compared to baseline data.
Wenclewska et al., 2019 [77]	Randomized controlled trial; Poland	T2D patients, 92	48; 14 (29)	63.43 ±1.57	2000 IU of vitamin D3 per day for three months.	Vitamin D supplementation increased HDL compared to baseline data.
Angellotti et al., 2019 [60]	Randomized, double-blind, placebo-controlled clinical trial; USA	T2D patients, 114	66; 49 (71)	60.1 ± 8.4	4000 units of vitamin D3 for 48 weeks.	Vitamin D supplementation revealed no significant in CRP, with a significant reduction in TG levels.
Fazelian et al., 2018 [51]	Randomized double-blind placebo-controlled clinical trial; Iran	T2D patients, 51	26; 0 (0)	48.5 ± 7.58	one oral pearl of 50,000 IU vitamin D3 for 16 weeks.	Vitamin D supplementation significantly reduced CRP and increased IL-10 levels.
Upreti et al., 2018 [70]	Parallel randomized, placebo-controlled trial; India	T2D patients, 60	30; 15 (50)	48.3 ± 9.8	60,000 IU weekly for six weeks, followed by once every four weeks for 24 weeks.	Vitamin D supplementation led to a significant difference in total cholesterol compared to the placebo group.
Barchetta et al., 2016 [64]	Randomized, double-blind, placebo-controlled trial; Italy	T2D patients, 65	26; 18 (50)	57.4 ± 10.7	2000 IU cholecalciferol per day for 24 weeks.	Vitamin D supplementation showed a significant difference in LDL, HDL, and TG without any significant difference in CRP levels.
Dalan et al., 2016 [74]	Parallel randomized, double-blind, placebo-con- trolled trial; Singapore	T2D patients, 61	31; 14 (45)	52.2 ± 8.2	4000 IU vitamin D (oral cholecalciferol) and 2000 IU for 16 weeks.	Vitamin D supplementation showed no significant effect on lipid profiles and CRP in comparison to baseline data.
Sadiya et al., 2015 [47]	Randomized, double-blind clinical trial; United Arab Emirates	T2D patients with obesity, 82.	43; 9 (20.9)	49 ± 8.0	Vitamin D (6000 IU) per day, followed by 3000 IU vitamin D3 daily for six months.	Vitamin D supplementation showed no significant differences in lipids and CRP compared to placebo groups.
Muñoz-Aguirre et al., 2014 [73]	Randomized, double-blind, placebo- controlled trial; Mexico	T2D patients, 104.	52; 0 (0)	56.1 ± 5.1	4000 IU of vitamin D daily for six months.	Vitamin D supplementation revealed no significant changes in LDL, and TC levels significantly decreased TG compared to the placebo.
Gagnon et al., 2014 [67]	Randomized, double-blinded, placebo-controlled trial; Australia	T2D patients, 80.	35; 10 (28.6)	53.8 ± 11.9	2000 IU of vitamin D3 for six months.	Vitamin D supplementation showed no difference in inflammatory markers compared to placebo.
Jehle et al., 2014 [75]	Prospective, randomized, double-blind, placebo-con- trolled pilot; Switzerland	T2D patients, 55.	29; 10 (34.5)	66.9 ± 3.1	A single 300,000 IU intramuscular injection of vitamin D3 for six months.	Vitamin D supplementation revealed no significant difference in levels of CRP compared to placebo groups.
Kampmann et al., 2014 [69]	Randomized, double-blind, placebo-controlled trial; Denmark	T2D patients, 16.	8; 6 (75)	61.6 ± 4.4	11,200 IU cholecalciferol per day for ten weeks	Vitamin D supplementation resulted in no significant difference in HDL, LDL, TC, TG, CRP, TNF-α, IL-6, and IL-8 compared to placebo groups.
Maggi et al., 2014 [66]	Randomized, double-blind, placebo-controlled clinical trial; Italy	T2D patients, 30	14; 9 (64)	69 ± 4.5	Single oral dose of 300,000 IU of Vitamin D3 for 24 weeks.	Vitamin D supplementation led to no significant difference in TNF-α levels compared to the group on placebo.
Ryu et al., 2014 [72]	Prospective, randomized, double-blind- ed, placebo-controlled trial; Korea	T2D patients, 158	79; NR	54.8 ± 7.6	1000 IU of vitamin D3 with a combined 100 mg of calcium twice daily for 24 weeks.	Vitamin D supplementation showed no significant difference in lipid profiles and inflammatory markers compared to placebo groups.
Tabesh et al., 2014 [50]	Parallel- randomized placebo- controlled clinical trial; Iran	T2D patients, 118	29; 15 (51.7)	50.2 ± 6.6	50,000 IU vitamin D3 per week for eight weeks.	Vitamin D supplementation showed no significant effect on serum levels of HDL, LDL, and TG compared to a placebo group.
Akbarzadeh et al., 2013 [49]	Randomized double-blind placebo-controlled trial; Iran	T2D patients, 70	35; 35 (100)	53.8 ± 8.9	Two tablets of Calcitriol (0.25 μg 1,25-dihydroxy cholecalciferol) (≈10 IU) per day for 12 weeks.	Vitamin D supplementation showed no significant effect on the marker of inflammation, including CRP, IL-6, and IL-18 levels, compared to baseline data.
Breslavsky et al., 2013 [71]	Randomized, double-blind, placebo-controlled trial; Israel	T2D patients, 47	24; 11 (45.8)	66.8 ± 9.2	1000 IU Vitamin D daily for 12 months.	Vitamin D supplementation had no significant effect on lipid profile and CRP compared to the placebo group.
Neyestani et al., 2012 [48]	Randomized, double-blinded controlled trial; Iran	T2D Patients, 60	30; NR.	51.5 ± 5.4	500 IU vitamin D3 and 150 calcium for 12 weeks.	Vitamin D supplementation significantly decreased CRP, IL-1β, and IL-6 compared to placebo.
Punthakee et al., 2012 [62]	Randomized, double-blind placebo-controlled trial; Canada	T2D patients, 1332	607; 362 (59.6)	66.7 ± 6.7	1000 IU daily for five and a half years.	Vitamin D supplementation showed no significant effect on HDL, LDL, TG, and TC in comparison to placebo.
Witham et al., 2010 [61]	Parallel, randomized, placebo- controlled trial; Canada	T2D patients, 95	37; 13 (35)	64.27 ± 10.27	A single dose of 100,000 IU vitamin D3 or 200,000 IU vitamin D3 for 16 weeks.	There was no significant difference in TC levels between the vitamin D and placebo groups.

T2D: type 2 diabetes, RCT: randomized controlled trial, HDL: high-density lipoprotein, LDL: low-density lipoprotein, TNF-α: tumor necrosis factor-alpha, IL-6: interleukin 6, CRP: C-reactive protein.

### 3.3. The Methodological Quality of Included RCTs

The overall quality of included RCTs was excellent, with a JADAD median and range score of 4 (1–5). Based on the domains, randomization was rated as 2 (1–2), blinding 1.5 (0–2), and the account of all patients was 1 (0–1). Additional details about the methodological quality of these RCTs are presented in Supplementary Table S2.

### 3.4. Effect of Vitamin D on Markers of Inflammation

#### 3.4.1. Effect of Vitamin D on High Sensitivity-C-Reactive Protein (hs-CRP) in T2D Patients

**Figure 2:**
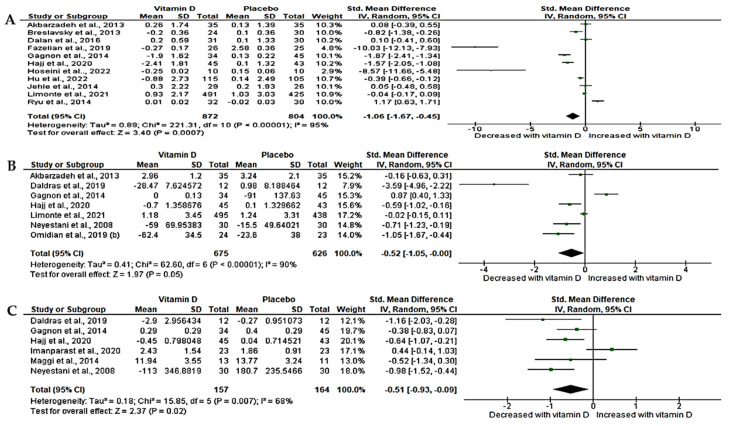
Effect of vitamin D on pro-inflammatory markers, (**A**) high sensitivity-C-reactive protein, (**B**) Interleukin-6, (**C**) Tumor necrosis factor-α in T2D patients compared to matched-placebo. Std: standard mean difference, CI: confidence intervals. Data are reported as standard mean difference and 95% confidence intervals with a *p*-value set at <0.05. Akbarzadeh et al., 2013 [49]; Breslavsky et al., 2013 [71]; Dalan et al., 2016 [74]; Daldras et al., 2019 [54]; Fazelian et al., 2018 [51]; Gagnon et al., 2014 [67]; Hajj et al., 2020 [76]; Hoseini et al., 2022 [57]; Hu et al., 2022 [68]; Jehle et al., 2014 [75]; Limonte et al., 2021 [58]; Ryu et al., 2014 [72]; Neyestani et al., 2008 [48]; Omidian et al., 2019 (b) [53]; Maggi et al., 2014 [66]; Imanparast et al., 2020 [56].

The level of hs-CRP was determined in 11 RCTs [49,51,58,67,68,71,72,74,75,76,78] with a sample size of 1676. The overall changes in hs-CRP were either extracted or estimated from baseline and post-treatment results. The analyzed data revealed a significant effect of vitamin D on hs-CRP in T2D patients compared to placebo. This is demonstrated by a significant decrease in the level of circulating hs-CRP (SMD = −1.06, 95%CI (−1.67, −0.45); *p* < 0.05). We observed a significant statistical heterogeneity amongst the studies (*I*^2^ = 95%, *p* < 0.05) (Figure 2A).

#### 3.4.2. Effect of Vitamin D on Interleukin-6 (IL-6)

We found that only six relevant trials [48,49,53,54,58,67,76] with a sample size of 1301 determined the level of IL-6 following vitamin D supplementation in T2D. Our results showed the beneficial effects of vitamin D on IL-6, which was demonstrated by a marginal decrease in its circulating levels (SMD= −0.52, 95%CI (−1.05, 0.01, *p* = 0.05). However, statistical heterogeneity was noted across these RCTs (*I*^2^ = 90%, *p* < 0.05) (Figure 2B).

#### 3.4.3. Effect of Vitamin D on Tumor Necrosis Factor-Alpha (TNF-α)

The inflammatory biomarker, TNF-α, was determined in 6 trials [48,54,56,66,67,76] with a sample size of 321. Of interest is that vitamin D exhibited potential benefits on TNF-α as shown by a significant decrease in TNF-α when compared to placebo (SMD = −0.51, 95%CI (−0.93, −0.09); *p* = 0.02). However, moderate statistical heterogeneity was observed (*I*^2^ = 68%, *p* < 0.05) (Figure 2C).

### 3.5. Effect of Vitamin D on Lipid Profiles (Triglycerides and Total Cholesterol)

**Figure 3:**
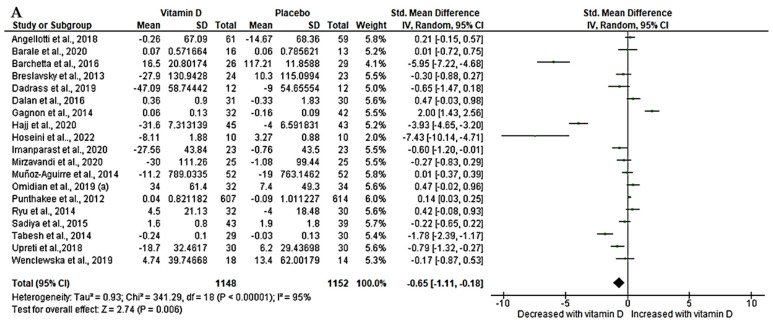

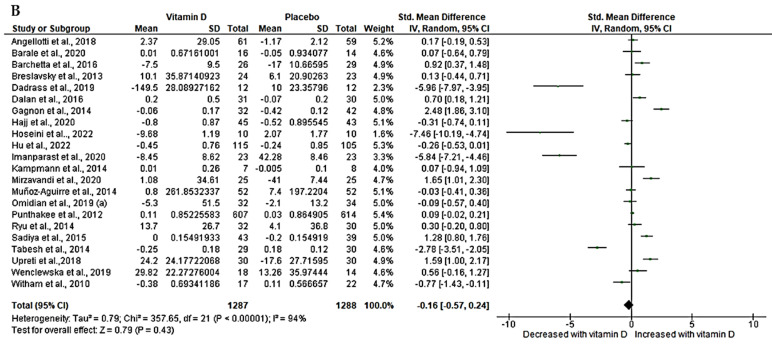
Effect of vitamin D on lipid profile, (**A**) triglycerides, (**B**) total cholesterol in type 2 diabetes. Std: standard mean difference, CI: confidence intervals. Data are reported as standard mean difference and 95% confidence intervals with a *p*-value set at *p* < 0.05. Angellotti et al., 2018 [60]; Barale et al., 2020 [65]; Barchetta et al., 2016 [64]; Breslavsky et al., 2013 [71]; Dadras et al., 2019 [54]; Dalan et al., 2016 [74]; Gagnon et al., 2014 [67]; Hajj et al., 2020 [76]; Hoseini et al., 2022 [57]; Hu et al., 2022 [68]; Imanparast et al., 2020 [56]; Kampmann et al., 2014 [69]; Mirzavandi et al., 2020 [55]; Muñoz-Aguirre et al., 2014 [73]; Omidian et al., 2019 (a) [52]; Punthakee et al., 2012 [62]; Ryu et al., 2014 [72]; Sadiya et al., 2015 [47]; Tabesh et al., 2014 [50]; Upreti et al., 2018 [70]; Wenclewska et al., 2019 [77]; Withan et al., 2010 [61].

From our relevant trials, only 19 [47,50,52,54,55,56,57,60,62,64,65,67,70,71,72,73,74,76,77] with a sample size of 2300 that determined triglycerides had enough data to be pooled for meta-analysis. Our effect estimates demonstrated a significant effect of vitamin D on triglyceride. This is revealed by a significant decrease in triglyceride (SMD = −0.65, 95%CI (−1.11, −0.18), *p* < 0.05) with a high level of statistical heterogeneity (*I*^2^ = 95%, *p* < 0.05) (Figure 3A). In contrast, total cholesterol was reported in 22 trials [47,50,52,54,55,56,57,60,62,64,65,67,68,70,71,72,73,74,76,77] with a sample size of 2575 between the two groups. Our pooled evidence shows that vitamin D had no significant statistical difference in total cholesterol when T2D patients were given either vitamin D or matched placebo drugs. However, a moderate decrease was observed (SMD = −0.16, 95%CI (−0.57, 0.24), *p* = 0.43) accompanied by a significant heterogeneity (*I*^2^ = 94%, *p* < 0.05) (Figure 3B).

### 3.6. Effect of Vitamin D on Lipid Profiles (HDL and LDL)

**Figure 4:**
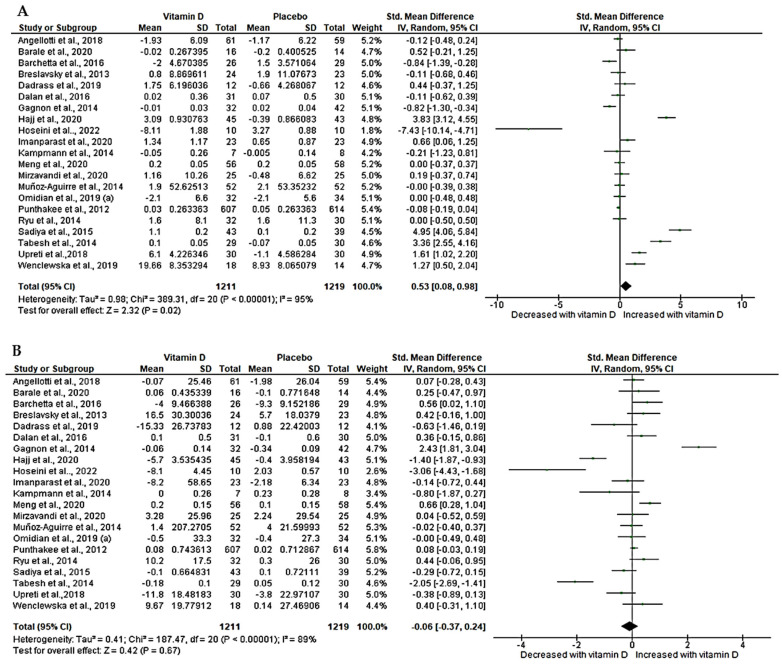
Effect of vitamin D on lipid profiles, (**A**) high-density lipoprotein (HDL), (**B**) low-density lipoprotein (LDL). Std: standard mean difference, CI: confidence intervals. Data are reported as standard mean difference and 95% confidence intervals with *p* set at *p* < 0.05. Angellotti et al., 2018 [60]; Barale et al., 2020 [65]; Barchetta et al., 2016 [64]; Breslavsky et al., 2013 [71]; Dadrass et al., 2019 [54]; Dalan et al., 2016 [74]; Gagnon et al., 2014 [67]; Hajj et al., 2020 [76]; Hoseini et al., 2022 [57]; Imanparast et al., 2020 [56]; Kampmann et al., 2014 [69]; Meng et al., 2020 [59]; Mirzavandi et al., 2020 [55]; Muñoz-Aguirre et al., 2014 [73]; Omidian et al., 2019 (a) [52]; Punthakee et al., 2012 [62]; Ryu et al., 2014 [72]; Sadiya et al., 2015 [47]; Tabesh et al., 2014 [50]; Upreti et al., 2018 [70]; Wenclewska et al., 2019 [77].

High-density lipoprotein was determined in 21 trials [47,50,52,54,55,56,57,59,60,62,64,65,67,70,71,72,73,74,76,77] with a sample size of 2430. Our pooled effect estimates showed a significant increase in HDL following vitamin D treatment in T2D patients compared to matched placebo (SMD = 0.53, 95%CI (0.08, 0.98), *p* = 0.02). Of concern was a high statistical heterogeneity amongst these trials (*I*^2^ = 95%, *p* < 0.05) (Figure 4A). Similarly, low-density lipoprotein was reported in 21 trials [47,50,52,54,55,56,57,59,60,62,64,65,67,70,71,72,73,74,76,77] with a sample size of 2430. Our analysis showed a slight decrease in LDL levels; however, this was not statistically significant (SMD = −0.06, 95CI (−0.37, 0.24), *p* = 0.67, *I*^2^ = 89%, *p* < 0.05) (Figure 4B).

### 3.7. Subgroup Analysis

Subgroup analysis was conducted for all effect measures based on gender distribution dose and duration of vitamin D intervention. We classified the doses as low or high, the intervention duration as short- or long-term, and gender as male, female, both, or unreported. The overall results are presented in the Supplementary File as Table S3. Briefly, our results showed no significant changes in heterogeneity (*I*^2^ remained constant or changed minimally) following subgroup analysis. For instance, the dosage, duration, and gender subgroup test revealed no major change in heterogeneity (*I*^2^ > 50%) on hs-CRP (Supplementary Table S3). For IL-6, the subgroup on dosage and gender distribution revealed minimal heterogeneity (*I*^2^ = 31.2%) and (*I*^2^ = 13.9%), respectively (Supplementary Table S3). Test for subgroup analysis on TNF-α showed a decrease in heterogeneity following subgroup on dosage and gender (Supplementary Table S3). Interestingly, the subgroup based on the duration of intervention revealed no evidence of heterogeneity (*I*^2^ = 0%), which was associated with the longer duration of vitamin D supplementation (Supplementary Table S3). Following subgroup analysis, on duration of intervention, a longer duration showed no evidence of heterogeneity in total cholesterol (*I*^2^ = 0%) and triglyceride (*I*^2^ = 0%) (Supplementary Table S3). Similarly, HDL and LDL duration and dosage seem to reduce heterogeneity to minimal, with long vitamin D supplementation resulting in zero heterogeneity (*I*^2^ = 0%) (Supplementary Table S3).

### 3.8. Sensitivity Analysis

Sensitivity was performed using the leave-one-study-out approach to evaluate the stability of the effect size across all outcomes. Results are presented in Supplementary Table S4–S10. For hs-CRP, exclusion of a trial by Fazelian [51] due to low weight led to SMD = −0.58, 95%CI (−0.19, −0.24), *p* = 0.03, *I*^2^ = 94% (Supplementary Table S4). We found that for IL-6, removing one study [54] due to the small sample size resulted in a change in effect size from original to SMD = −0.25, 95%CI (−0.68, 0.18), *p* = 0.26, *I*^2^ = 87% (Supplementary Table S5), for TNF-α, exclusion of [54] resulted in SMD = −0.42, 95%CI (−0.87, 0.02), *p* = 0.06, *I*^2^ = 70.5% (Supplementary Table S6). For triglycerides and total cholesterol, a study with a small sample size was excluded [57], and the effect size changed to (SMD = −0.06, 95%CI (0.20, −0.32), *p* = 0.77, *I*^2^ = 94%) (Supplementary Table S7) and SMD = −0.04, 95%CI (−0.43, 0.36), *p* = 0.85, *I*^2^ = 94% (Supplementary Table S8) respectively. Lastly, when the same study [57] was excluded for both HDL and LDL, there was a change in effect size SMD = 0.68, 95%CI (0.39, 0.97), *p* < 0.05, *I*^2^ = 95% (Supplementary Table S9) and SMD = 0.02 (−0.17, 0.21), *p* = 0.91, *I*^2^ = 89% (Supplementary Table S10), respectively.

### 3.9. Publication Bias

The publication bias was assessed visually and graphically through a funnel plot symmetrical shape (Supplementary Figures S1 and S2). For inflammatory markers, asymmetrical plot visualization was noted on IL-6 and hs-CRP, which suggests evidence of publication bias (Supplementary Figure S1A,B). The Eggers regression test further supports this, IL-6 (Z score = −3.21, *p* < 0.05), hs-CRP (Z-score = −7.74, *p* < 0.05). Interestingly, there was no evidence of publication bias on hs-CRP and TNF-α graphically (Supplementary Figure S1C) and statistically TNF-α (Z score = −0.49, *p* = 0.62). The visual examination of the funnel plot indicates the presence of publication bias for lipid profiles (Supplementary Figure S2A–D). This was also corroborated by Egger’s regression test total cholesterol (Z score = −6.84, *p* < 0.05), triglycerides (Z score = −5.88, *p* < 0.05), HDL (Z score = −2.23, *p* = 0.03) and LDL (Z score = −2.31, *p* = 0.02). One of the factors contributing to the observed publication bias is the disproportionate publication of studies from Iran compared to other countries. This can be attributed to the high prevalence of vitamin D deficiency among the Iranian population, suggesting a reliance on vitamin D supplementation in this region as opposed to other countries.

### 3.10. Certainty of Evidence

We further evaluated certainty by GRADING evidence gathered in this review using GRADEprofiler. The results are presented in Supplementary Table S11. In brief, our results were rated high-quality for TNF-α, IL-6, triglyceride, and total cholesterol, moderate for CRP and HDL, and low for LDL.

## 4. Discussion

We gathered evidence from 30 RCTs to simultaneously evaluate the effect of vitamin D on inflammatory markers and lipid profiles in type 2 diabetic patients. This meta-analysis included trials with at least 2310 T2D patients on vitamin D supplementation and revealed vitamin D’s potential to ameliorate inflammation. This is demonstrated by a significant reduction in circulating hs-CRP and TNF-α. A marginal potential effect was also observed on IL-6 following vitamin D supplementation. These results suggest that vitamin D supplementation in T2D may be beneficial in alleviating inflammatory-associated complications. Moreover, our findings are supported by Gu et al., 2022 [79]. Our results are corroborated by Calton et al., 2015, who demonstrated an anti-inflammatory effect of vitamin D through evidence from an in vitro study [80]. These researchers assessed monocyte chemoattractant protein-1 (MCP-1), IL-6, and IL-8 as markers of inflammation, which were reduced after 1,25(OH)2, cholecalciferol, and 25(OH)D treatment. This meta-analysis demonstrated the ameliorative effect of vitamin D on inflammation by reducing the level of CRP and TNF-α. Notably, our study indicated a marginal decrease in IL-6 (*p* = 0.05) in T2D following vitamin D supplementation. However, it is noteworthy that a prior meta-analysis by Yu et al., 2018, revealed no significant effect of vitamin D supplementation on TNF-α and IL-6 despite supporting our findings on the observed significant decrease in hs-CRP [31]. Despite being conducted in T2D, a limitation of the prior meta-analysis lies in its sample size, comprising only 13 studies. Furthermore, the intervention group encompassed studies using combined supplementation, such as calcium and vitamin K, which could influence the overall efficacy of vitamin D. For instance, Jorde has reported a negative correlation between vitamin D and calcium intake with the serum level of vitamin D [81].

Conversely, another study showed no significant effect of vitamin D on CRP and TNF-α serum levels. However, the same study revealed a significant increase in the serum IL-6 [82]. Increased IL-6 observed in this study implies that vitamin D promoted inflammation. This further suggests that vitamin D exhibited no anti-inflammatory effects among the included studies. These conflicting results may be attributed to a different form of vitamin D, dosage, duration of intervention, and the main aspect in terms of population, as evidence was pooled from studies where patients had T2D, human immune-deficiency virus (HIV), pre-diabetes, and non-alcoholic fatty liver diseases. For example, HIV independently is associated with chronic inflammation [83]. Also, non-alcoholic fatty liver disease is an inflammation of the liver due to fat accumulation in the hepatocytes [84]. An existing meta-analysis by Chen and his team in 2014 reported a significant decrease in CRP levels following vitamin D administration [85]. Notably, this meta-analysis evaluated a group of patients with various metabolic conditions ranging from obesity, pregnant women, T2D, coronary artery disease, polycystic ovary syndrome, insulin-resistant condition, or bedridden older patients in addition to healthy participants contrary to our focus, which is strictly on T2D. All these conditions independently may trigger an inflammatory response, and thus, the effect observed might not necessarily be due to vitamin D but be attributed to these conditions. Another qualitative study synthesized by Agbalalah et al., 2017 [86], reported no benefit of vitamin D supplementation in adult patients, and this study was based on a wide range of conditions, including HIV, chronic kidney disease (CKD), and T2D. Although this study reported null findings, this can be due to the fact that HIV is associated with increased immune activation, ongoing HIV replication, and immune dysfunction, which contributes to chronic states of inflammation [83].

Similarly, CKD activates NF-κβ and Toll-like receptor pathways, producing inflammatory molecules that exacerbate inflammation [87]. Hence, our results suggest that vitamin D can inhibit the production of pro-inflammatory markers, including TNF-α, IL-6, and hs-CRP. These markers promote an inflammatory response. Therefore, vitamin D, by reducing their production, may ameliorate inflammation. Some of the mechanisms by which vitamin D regulates inflammation are mediated by signaling pathways, including cyclooxygenase suppression of NF-κβ. These regulate inflammatory gene expression and mitogen-activated protein kinase (MAPK) activation, mediating inflammatory responses [88]. Therefore, by inhibiting p38 MAPK, vitamin D can suppress the production of pro-inflammatory cytokines, such as TNF-α and IL-6 in macrophages [89]. Inhibition of p38 gene expression in macrophages activates MAPK phosphatase-1 (MKP1), which dephosphorylates p38 and thus reduces p38 activation. Vitamin D and its receptor complex can interact with NF-κβ, or the glucocorticoid receptor, which results in anti-inflammatory effects [88]. Likewise, vitamin D modulates T-cells through its receptors. This action inhibits the differentiation of T-cells into pro-inflammatory subsets and thus promotes the development of regulatory T-cells, which exhibit anti-inflammatory properties [90,91].

Secondly, in terms of the effects of vitamin D on lipid profiles, we found that vitamin D administration can ameliorate dyslipidemia, as shown by a significant decrease in triglycerides, concomitant to an increased HDL. Increased triglyceride levels in the body are associated with atherosclerosis and increased risk of heart failure. Our findings are supported by these trials [55,60,63,64,73,77]. However, some studies disagree with our findings despite being conducted in T2D [56,59,62,69,71,72,92,93]. It is important to note that reducing triglycerides and increasing HDL, especially in T2D patients, may curb secondary complications associated with T2D. Although there was a notable reduction in total cholesterol and LDL in our current study, this was statistically not significant in the current analysis. These findings are partly supported by Qi et al., 2022, who reported no effect of vitamin D on lipid profile in general; however, this meta-analysis was conducted in patients with metabolic syndromes [94] and might not be comparable to our findings. This is partly due to different underlying mechanisms, severity, individual responses, and management approaches.

The ameliorative effect of vitamin D on cholesterol levels is attributable to its potential to influence insulin-gene expression, the transcription activity on vitamin D receptor (VDR), downregulate activation of sterol regulatory element binding protein-2 (SREBP-2), inhibition of 3-hydroxy-3-methyl glutaryl-coenzyme-A reductase expression, a cofactor for the synthesis of cholesterol with subsequent reduction in cholesterol level [95]. Preclinical evidence also corroborates findings observed in clinical studies by inhibiting SREBP-2 expression [96]. Another animal study also demonstrated vitamin D’s role in maintaining lipid profile by regulating lipogenic genes through downregulating SREBP [97]. In an experimental study, active vitamin D reduced triglycerides in differentiated adipocytes, increased fatty acid β-oxidation, and reduced de novo fatty acid synthesis [98].

Despite the well-designed method used in this systematic review and meta-analysis, the limitations remain; for example, this study included all trials despite different doses and duration of the intervention. Secondly, there was high heterogeneity across the included trials; however, subgroup analyses according to these confounding factors, such as dosage, duration, and gender, proved to have no significant effect on other parameters except TNF-α, total cholesterol, and HDL (*I*^2^ = 0%). In addition, sensitivity analysis was performed to evaluate the stability of our effect size and revealed no major changes except a study with a small sample size across all outcome measures. Of interest with this current study is that the sample size used was sufficient as about 30 trials with 2310 T2D patients on vitamin D were included in a meta-analysis. This is a powered sample size and sufficient to make a conclusive statement and recommendation about the efficacy of vitamin D as an anti-inflammatory and anti-dyslipidemic agent in adults with T2D. The overall quality of the included trial was excellent (83%), although two trials individually across all domains were poor (7%), and three were fair (10%) in overall quality. Our certainty of the evidence was high for IL-6, TNF-α, triglyceride, and total cholesterol, moderate for both hs-CRP and HDL, and low for LDL.

Furthermore, the methodology followed in this study was rigorous and adhered to established guidelines of PRISMA. To avoid potential inconsistencies and biases in the selection, extraction, quality assessment, and evidence grading, a minimum of three researchers were involved in each phase. By involving multiple researchers, we ensured that the findings were reliable, thus strengthening the overall validity of the outcomes of this study.

## 5. Conclusions and Future Perspectives

The evidence obtained in this meta-analysis provides valuable insights into the potential benefits of vitamin D supplementation among T2D patients. Specifically, the results suggest that vitamin D supplementation can have anti-inflammatory and anti-hyperlipidemic effects on T2D. After supplementation, a reduction in TNF-α, IL-6, and hs-CRP levels demonstrates its anti-inflammatory effect, while the decrease in triglycerides and increase in HDL levels demonstrate its anti-hyperlipidemic properties. However, it is important to note that while such benefits were observed, this study found no statistically significant effect of vitamin D supplementation on total cholesterol and LDL amongst these adult T2D patients. This suggests that vitamin D supplementation might not significantly impact these lipid parameters in T2D. The results also raise interesting questions for future research, which needs to focus on proper methodology and powered sample size trials to further investigate the effects of vitamin D supplementation on lipids profiles in T2D patients.

Moreover, exploring different doses of vitamin D, at high and low, with different duration of intervention could provide insights into the optimal dosing and duration of vitamin D for maximum benefits in T2D. Furthermore, the safety of vitamin D should be considered when exploring the long-term effects in this population. Therefore, future trials should also focus on assessing the safety profile of different doses and durations of vitamin D supplementation to ensure that it does not cause adverse effects, especially when administered over extended periods.

## Data Availability

All data supporting this manuscript is supplied in the form of a Supplementary File.

## Supplementary Materials

The following supporting information can be downloaded at https://www.mdpi.com/article/10.3390/nu15214575/s1, Figure S1: Funnel plots showing bias on inflammatory markers.; Figure S2: Funnel plots showing bias on lipid profile.; Table S1: PubMed Search strategy was updated on the 21 June 2023; Table S2: Quality assessment according to JADAD guidelines; Table S3: Subgroup analysis of RCTs according to dosage, duration of intervention and gender distribution. Table S4: Sensitivity analysis for hs-CRP using leave-one analysis; Table S5: Sensitivity analysis using leave one analysis for IL-6; Table S6: Sensitivity analysis using leave one for TNF-alpha; Table S7: Sensitivity analysis for TG using leave-one analysis; Table S8: Sensitivity analysis for TC using leave-one analysis; Table S9: Sensitivity analysis for HDL using leave-one analysis; Table S10: Sensitivity analysis for LDL using leave-one analysis; Table S11: Summary of findings table to evaluate certainty of evidence.

## Abbreviations

CI	Confidence intervals.
CRP	C-reactive protein.
CVD	Cardiovascular disease.
GRADE	Grading of Recommendation Assessment, Development, and Evaluation.
HDL	High-density lipoprotein.
IDF	International Diabetes Federation.
IFγ	Interferon-gamma.
IL-6	Interleukin-6.
IQR	Interquartile Range.
IU	International Unit.
LDL	Low-density lipoprotein.
MCP-1	Monocyte-Chemoattractant Protein-1.
MD	Mean difference.
NF-kβ	Nuclear Factor kappa beta.
NO	Nitric oxide.
NR	Not Reported.
oxLDL	Oxidised Low-density lipoprotein.
PRISMA	Preferred Reporting Items for Systematic Review and Meta-analysis.
RM	Randomized Model.
SD	Standard deviation.
SEM	Standard Error of Mean.
SMD	Standard mean difference.
T2D	Type 2 diabetes.
TNF-α	Tumor necrosis factor-alpha.
