# Supplementary material for: Modulatory Properties of Vitamin D in Type 2 Diabetic Patients: A Focus on Inflammation and Dyslipidemia
Source: Nutrients. 2023 Oct 27;15(21):4575. doi: 10.3390/nu15214575 (PMC10650901; doi:10.3390/nu15214575)
Supplement: Supplementary file 1 [file nutrients-15-04575-s001.zip › nutrients-2685599-supplementary.pdf]

*Systematic Review*

# Modulatory Properties of Vitamin D in Type 2 Diabetic Patients: A Focus on Inflammation and Dyslipidemia

**Rizqah MacGirlley, Wendy N. Phoswa and Kabelo Mokgalaboni ***

Department of Life and Consumer Sciences, College of Agriculture and Environmental Sciences, University of South Africa, Florida Campus, Private Bag X6, Roodepoort 1710, South Africa; 10916970@mylife.unisa.ac.za (R.M.); phoswwn@unisa.ac.za (W.N.P.)
* Correspondence: mokgak@unisa.ac.za

**Table S1:** PubMed Search strategy was updated on the 21 of June 2023

| Item | MeSH terms | Search | Hits |
|---|---|---|---|
| 1 | Vitamin D | Vitamin D[MeSH Terms] Filters: Randomized Controlled Trial, from 1000/1/1 - 2023/6/21 | 4231 |
| 2 | 1,25-Dihydroxycholecalciferol | 1,25-Dihydroxycholecalciferol[MeSH Terms] Filters: Randomized Controlled Trial, from 1000/1/1 - 2023/6/21 | 675 |
| 3 | Calciferol | Calciferol[MeSH Terms] Filters: Randomized Controlled Trial, from 1000/1/1 - 2023/6/21 | 290 |
| 4 | Type 2 diabetes mellitus | Type 2 diabetes mellitus[MeSH Terms] Filters: Randomized Controlled Trial, from 1000/1/1 - 2023/6/21 | 14331 |
| 5 | 1,2,3 and 4 | (((Vitamin D[MeSH Terms] AND ((randomizedcontrolledtrial[Filter]) AND (1000/1/1:2023/6/21[pdat]))) OR (1,25-Dihydroxycholecalciferol[MeSH Terms] AND ((randomizedcontrolledtrial[Filter]) AND (1000/1/1:2023/6/21[pdat])))) OR (Calciferol[MeSH Terms] AND ((randomizedcontrolledtrial[Filter]) AND (1000/1/1:2023/6/21[pdat])))) AND (Type 2 diabetes mellitus[MeSH Terms] AND ((randomizedcontrolledtrial[Filter]) AND (1000/1/1:2023/6/21[pdat]))) Filters: Randomized Controlled Trial, from 1000/1/1 - 2023/6/21 | 172 |

**Table S2**: Quality assessment according to JADAD guidelines.

| **Study** | **Descriptions** | | | | |
|---|---|---|---|---|---|
| | **Randomization** | **Blinding** | **An account of all patien** | **Total score** | **Rating** |
| Hoseini et al., 2022 [11] | 2 | 1 | 1 | 4 | Excellent |
| Hu et al., 2022 [21] | 2 | 1 | 1 | 4 | Excellent |
| Limonte et al., 2021 [12] | 1 | 2 | 1 | 4 | Excellent |
| Barale et al., 2020 [18] | 2 | 1 | 1 | 4 | Excellent |
| Hajj et al., 2020 [29] | 1 | 1 | 1 | 3 | Fair |
| Imanparast al., 2020[10] | 1 | 0 | 0 | 1 | Poor |
| Meng et al., 2020 [13] | 1 | 2 | 1 | 4 | Excellent |

| | | | | | |
|---|---|---|---|---|---|
| Mirzavandi et al., 2020[9] | 2 | 1 | 1 | 4 | Excellent |
| Angellotti et al., 2019[14] | 2 | 1 | 1 | 4 | Excellent |
| Dadrass et al., 2019 [8] | 2 | 2 | 1 | 5 | Excellent |
| Omidian et al 2019 (a)[6] | 2 | 2 | 1 | 5 | Excellent |
| Omidian et al 2019 (b) [7] | 2 | 2 | 1 | 5 | Excellent |
| Wenclewska et al., 2019 [30] | 1 | 0 | 1 | 2 | Poor |
| Fazelian et al., 2018[5] | 2 | 2 | 1 | 5 | Excellent |
| Upretti et al., 2018[23] | 2 | 2 | 1 | 5 | Excellent |
| Dalan et al. 2016[27] | 2 | 2 | 1 | 5 | Excellent |
| Barchetta et al., 2016[17] | 2 | 1 | 1 | 4 | Excellent |
| Sadiya et al., 2015 [1] | 2 | 2 | 1 | 5 | Excellent |
| Gagnon et al., 2014 [20] | 2 | 2 | 1 | 5 | Excellent |
| Jehle et al.,2014[28] | 2 | 2 | 1 | 5 | Excellent |
| Kampmann et al.,2014 [22] | 2 | 2 | 1 | 5 | Excellent |
| Muñoz-Aguirre et al., 2014[26] | 2 | 1 | 1 | 4 | Excellent |
| Tabesh et al., 2014[4] | 2 | 2 | 1 | 5 | Excellent |
| Maggi et al., 2014[19] | 2 | 1 | 0 | 3 | Fair |
| Ryu et al.,2014[25] | 2 | 1 | 1 | 4 | Excellent |
| Akbarzadeh et al., 2013 [3] | 2 | 2 | 1 | 5 | Excellent |
| Breslavsky et al., 2013[24] | 2 | 1 | 1 | 4 | Excellent |
| Punthakee et al., 2012[16] | 2 | 1 | 1 | 4 | Excellent |
| Neyestani et al., 2012[2] | 1 | 1 | 1 | 3 | Fair |
| Witham et al., 2010[15]. | 2 | 2 | 1 | 5 | Excellent |
| **Average** | **1.8** | **1.4** | **0.9** | **4.2** | **Excellent** |
| **Median** | **2** | **1** | **1** | **4** | **Excellent** |

**Table S3:** Subgroup analysis of RCTs according to dosage, duration of intervention and gender distribution.

| **Effect measure** | **Number of studies** | **SMD, 95%CI** | **p** | **$I^2$** | **Subgroup Difference ($I^2$, p)** |
|---|---|---|---|---|---|
| **hs-CRP** | | | | | |
| **Dosage** | 11 | -0.93 [-1.51, -0.35] | <0.05 | 96 | |
| Low-dose | 7 | -0.25 [-0.72, 0.23] | 0.31 | 92 | 87.2%,<0.05 |
| High-dose | 4 | -2.91 [-4.71, -1.10] | <0.05 | 97 | |
| **Duration** | 11 | -1.06 [-1.67, -0.45] | <0.05 | 95 | |
| Short period | 8 | -1.88 [-3.07, -0.70] | <0.05 | 97 | 83.1%, 0.01 |
| Long period | 3 | -0.34 [-0.72, 0.04] | 0.08 | 82 | |

| | | | | | |
|---|---|---|---|---|---|
| **Gender** | 11 | -1.06 [-1.67, -0.45] | <0.05 | 95 | |
| Male | 2 | -4.10 [-12.57, 4.37] | 0.34 | 97 | |
| Female | 1 | -10.03[-12.13,7.93] | <0.05 | N/A | 97.3%, <0.05 |
| Both | 7 | -0.63 [-1.12, -0.14] | 0.01 | 93 | |
| Not reported | 1 | 1.17 [0.63, 1.71] | <0.05 | N/A | |
| | | | | | |
| **IL-6** | | | | | |
| **Dosage** | 7 | -0.52 [-1.05, -0.00] | 0.05 | 90% | |
| Low-dose | 5 | -0.18 [-0.68, 0.31] | 0.47 | 87% | 31.2%, 0.23 |
| High-dose | 2 | -2.02 [-4.96, 0.92] | 0.18 | 94% | |
| **Duration** | 7 | -0.52 [-1.05, -0.00] | 0.05 | 90 | |
| Short period | 6 | -0.71 [-1.46, 0.05] | 0.07 | 91 | 67.7%, 0.08 |
| Long period | 1 | -0.02 [-0.15, 0.11] | 0.67 | N/A | |
| **Gender** | 7 | -0.52 [-1.05, -0.00] | 0.05 | 90 | |
| Male | 2 | -1.82[-5.18, 1.55] | 0.29 | 95% | 13.9%, <0.05 |
| Both | 4 | -0.17[-0.78, 0.43] | 0.57 | 90% | |
| Not reported | 1 | -0.71[-1.23, -0.19] | 0.00 | N/A | |
| | | | | | |
| **TNF-$\alpha$** | | | | | |
| **Dosage** | 6 | -0.51 [-0.93, 0.09] | 0.02 | 68 | |
| Low-dose | 3 | -0.76 [-1.25, -0.27] | 0.00 | 51 | 27.7%, 0.24 |
| High-dose | 3 | -0.24 [-0.93, 0.49] | 0.52 | 77 | |
| **Duration** | 6 | -0.51 [-0.93, -0.09] | 0.02 | 68% | |
| Short period | 3 | -0.54 [-1.57, 0.49] | 0.30 | 87% | 0%, 0.96 |
| Long period | 3 | -0.51 [-0.80, -0.22] | 0.00 | 0% | |
| **Gender** | 6 | -0.51[-0.98, -0.09] | 0.02 | 68 | |
| Male | 1 | -1.16[-2.03, -0.28] | 0.01 | N/A | |
| Both | 4 | -0.28[-0.75, 0.19] | 0.24 | 66% | 60.6%, 0.08 |
| Not reported | 1 | -0.98[-1.52, -0.44] | 0.00 | N/A | |
| | | | | | |
| **Total cholesterol** | | | | | |
| **Dosage** | 22 | -0.37 [-0.82, 0.07] | 0.10 | 95 | |
| Low-dose | 14 | 0.21 [-0.18, 0.60] | 0.21 | 93 | 85.8%, 0.00 |
| High dose | 8 | -2.12 [-3.79, -0.44] | 0.01 | 97 | |
| **Duration** | 22 | -0.16 [-0.57, 0.24] | 0.43 | 94 | |
| Short period | 19 | -0.24 [-0.72, 0.25] | 0.33 | 95 | 0%, 0.78 |
| Long period | 3 | -0.16 [-0.39, 0.06] | 0.16 | 0 | |
| **Gender** | 22 | -0.24 [-0.73, 0.24] | 0.32 | 92 | |
| Male | 2 | -6.49 [-8.11, -4.87] | <0.05 | 0 | |
| Female | 17 | -0.03 [-0.41, 0.36] | 0.89 | N/A | 95.2%, <0.05 |
| Both | 2 | 0.06 [-0.45, 0.57] | 0.81 | 94 | |
| Not reported | 1 | 0.30 [-0.20, 0.80] | 0.25 | N/A | |
| **Triglycerides** | | | | | |
| **Dosage** | 19 | -0.65 [-1.11, -0.18] | 0.00 | 95 | |
| Low-dose | 12 | -0.05 [-0.48, 0.38] | 0.81 | 92 | 89%, 0.00 |
| High-dose | 7 | -1.81 [-2.87, -0.75] | 0.00 | 94 | |
| **Duration** | 19 | -065[-1.11,-0.19] | <0.05 | 95 | |
| Short | 17 | -0.73 [-1.23,-0.22] | <0.05 | 95 | 59.6%, 0.12 |
| Long | 2 | -0.18 [-0.63, 0.27] | 0.43 | 0 | |

| | | | | | |
|---|---|---|---|---|---|
| **Gender** | 19 | -0.84[-1.34, -0.34] | 0.00 | 95 | 74.5%, <0.05 |
| Male | 2 | -3.91[-10.55, 2.73] | 0.25 | 95 | |
| Female | 1 | 0.01[-0.37, 0.39] | 0.96 | N/A | |
| No reported | 1 | 0.42[-0.08, 0.93] | 0.10 | N/A | |
| Both | 15 | -0.85[-1.44, -0.26] | <0.05 | 96 | |
| | | | | | |
| **HDL** | | | | | |
| **Dosage** | 21 | 0.53[0.08, 0.98] | 0.02 | 95 | 0%, 0.52 |
| Low-dose | 13 | 0.26[-0.14, 0.67] | 0.20 | 92 | |
| High-dose | 8 | 0.69[-0.55, 1.92] | 0.28 | 95 | |
| **Duration** | 21 | 0.41[-0.07, 0.88] | 0.09 | 95 | 29.3%, 0.23 |
| Short | 17 | 0.37[-0.35, 1.09] | 0.31 | 98 | |
| Long | 4 | -0.07[-0.17, 0.04] | 0.20 | 0 | |
| **Gender** | 21 | 0.52[-0.00, 1.05] | 0.05 | 95 | 51.8%, 0.10 |
| Male | 2 | -3.38[-11.09, 4.32] | 0.38 | 97 | |
| Female | 1 | -0.00[-0.39, 0.38] | 0.90 | N/A | |
| Both | 17 | 0.80[0.19, 1.14] | 0.01 | 95 | |
| Not reported | 1 | 0.00[-0.50, 0.50] | 1.0 | N/A | |
| | | | | | |
| **LDL** | | | | | |
| **Dosage** | 21 | -0.20 [-0.46, 0.05] | 0.12 | 84 | 91.6%, <0.05 |
| Low-dose | 13 | 0.16 [-0.00, 0.31] | 0.06 | 45 | |
| High-dose | 8 | -0.96 [-1.58, -0.35] | <0.05 | 85 | |
| **Duration** | 21 | -0.06 [-0.37, 0.24] | 0.67 | 89 | 17.1%, 0.27 |
| Short | 17 | -0.16 [-0.62, 0.29] | 0.48 | 91 | |
| Long | 4 | 0.10 [-0.01, 0.20] | 0.07 | 0 | |
| **Gender** | 21 | -0.06 [-0.37, 0.24] | 0.67 | 89 | 36.4%, 0.19 |
| Male | 2 | -1.78 [-4.15, 0.59] | 0.14 | 89 | |
| Female | 1 | -0.02 [-0.40, 0.37] | 0.93 | N/A | |
| Both | 17 | 0.02 [-0.31, 0.36] | 0.89 | 90 | |
| Not reported | 1 | 0.44 [-0.06, 0.95] | 0.08 | N/A | |

**Table S4: Sensitivity analysis for hs-CRP using leave-one analysis**

| Deleted Study | Effect Size | Lower CI | Upper CI | P Value | I Square |
|---|---|---|---|---|---|
| Akbarzadeh et al., 2013 | -1.3105 | -1.7609 | -0.8601 | 0.0002 | 96.1454 |
| Breslavsky et al., 2013 | -1.1896 | -1.6306 | -0.7486 | 0.0005 | 96.0979 |
| Dalan et al., 2016 | -1.3054 | -1.7529 | -0.8580 | 0.0002 | 96.1468 |
| Fazelian et al., 2018 | -0.5757 | -0.9090 | -0.2423 | 0.0269 | 93.7778 |

| Deleted Study | Effect Size | Lower CI | Upper CI | P Value | I Square |
|---|---|---|---|---|---|
| Gagnon et al., 2014 | -1.0077 | -1.4189 | -0.5965 | 0.0017 | 95.4317 |
| Hajj et al., 2020 | -1.0634 | -1.4850 | -0.6419 | 0.0012 | 95.5774 |
| Hoseini et al., 2022 | -0.8336 | -1.2200 | -0.4472 | 0.0057 | 95.5210 |
| Hu et al., 2022 | -1.3587 | -1.8604 | -0.8570 | 0.0005 | 96.1454 |
| Jehle et al., 2014 | -1.2962 | -1.7422 | -0.8502 | 0.0002 | 96.1563 |
| Limonte et al., 2021 | -1.4787 | -2.0269 | -0.9306 | 0.0005 | 95.8358 |
| Ryu et al., 2014 | -1.3694 | -1.7914 | -0.9475 | 0.0000 | 95.6860 |

**Table S5: Sensitivity analysis using leave one analysis for IL-6**

| Deleted Study | Effect Size | Lower CI | Upper CI | P Value | I Square |
|---|---|---|---|---|---|
| **Akbarzadeh et al., 2013** | -0.6427 | -1.2791 | -0.0064 | 0.0477 | 92.3976 |
| **Daldras et al., 2019** | -0.2511 | -0.6841 | 0.1818 | 0.2556 | 86.7337 |
| **Gagnon et al., 2014** | -0.7824 | -1.3325 | -0.2322 | 0.0053 | 89.6063 |
| **Hajj et al., 2020** | -0.5615 | -1.1875 | 0.0646 | 0.0788 | 91.7384 |
| **Limonte et al., 2021** | -0.7257 | -1.4956 | 0.0441 | 0.0647 | 91.5404 |
| **Neyestani et al., 2008** | -0.5289 | -1.1277 | 0.0700 | 0.0835 | 91.7296 |
| **Omidian et al., 2019 (b)** | -0.4576 | -1.0222 | 0.1071 | 0.1122 | 91.1121 |

**Table S6: Sensitivity analysis using leave one for TNF-alpha**

| Deleted Study | Effect Size | Lower CI | Upper CI | P Value | I Square |
|---|---|---|---|---|---|
| **Daldras et al., 2019** | -0.4220 | -0.8669 | 0.0228 | 0.0630 | 70.5261 |
| **Gagnon et al., 2014** | -0.5496 | -1.0913 | -0.0080 | 0.0467 | 74.3225 |
| **Hajj et al., 2020** | -0.4873 | -1.0311 | 0.0565 | 0.0790 | 73.9833 |
| **Imanparast et al., 2020** | -0.6667 | -0.9225 | -0.4110 | 0.0000 | 6.6016 |
| **Maggi et al., 2014** | -0.5137 | -0.9999 | -0.0275 | 0.0384 | 74.9341 |
| **Neyestani et al., 2008** | -0.4093 | -0.8705 | 0.0519 | 0.0820 | 67.2507 |

**Table S7: Sensitivity analysis for TG using leave-one analysis**

| Deleted Study | Effect Size | Lower CI | Upper CI | P Value | I Square |
|---|---|---|---|---|---|
| Angellotti et al., 2018 | -0.2476 | -0.5379 | 0.0427 | 0.2744 | 94.7339 |
| Barale et al., 2020 | -0.2270 | -0.5079 | 0.0539 | 0.3005 | 94.7347 |
| Barchetta et al., 2016 | -0.2697 | -0.5500 | 0.0107 | 0.2176 | 94.6193 |
| Breslavsky et al., 2013 | -0.2334 | -0.5163 | 0.0495 | 0.2903 | 94.7349 |
| Dadrass et al., 2019 | -0.0414 | -0.3026 | 0.2198 | 0.8390 | 94.1002 |
| Dalan et al., 2016 | -0.2617 | -0.5440 | 0.0206 | 0.2349 | 94.6666 |
| Gagnon et al., 2014 | -0.3143 | -0.5753 | -0.0532 | 0.1229 | 93.7747 |
| Hajj et al., 2020 | -0.2143 | -0.4998 | 0.0713 | 0.3362 | 94.6749 |
| Hoseini et al.., 2022 | -0.0597 | -0.3215 | 0.2021 | 0.7701 | 94.1810 |
| Hu et al., 2022 | -0.2352 | -0.5324 | 0.0621 | 0.3106 | 94.6112 |
| Imanparast et al., 2020 | 0.0277 | -0.2213 | 0.2767 | 0.8866 | 93.3761 |
| Kampmann et al., 2014 | -0.2230 | -0.5017 | 0.0557 | 0.3051 | 94.7348 |
| Mirzavandi et al., 2020 | -0.2940 | -0.5680 | -0.0200 | 0.1691 | 94.4074 |
| Muñoz-Aguirre et al., 2014 | -0.2342 | -0.5228 | 0.0543 | 0.2982 | 94.7265 |
| Omidian et al., 2019 (a) | -0.2250 | -0.5097 | 0.0596 | 0.3109 | 94.7249 |
| Punthakee et al., 2012 | -0.3191 | -0.6638 | 0.0256 | 0.2354 | 94.7280 |
| Ryu et al., 2014 | -0.2442 | -0.5284 | 0.0400 | 0.2708 | 94.7282 |
| Sadiya et al., 2015 | -0.2836 | -0.5604 | -0.0069 | 0.1891 | 94.3888 |
| Tabesh et al., 2014 | -0.0502 | -0.3080 | 0.2075 | 0.8028 | 93.6678 |
| Upreti et al.,2018 | -0.2923 | -0.5662 | -0.0184 | 0.1714 | 94.3644 |
| Wenclewska et al., 2019 | -0.2496 | -0.5301 | 0.0309 | 0.2540 | 94.7147 |
| Witham et al., 2010 | -0.1826 | -0.4617 | 0.0965 | 0.4017 | 94.6334 |

**Table S8: Sensitivity analysis for TC using leave-one analysis**

| Deleted Study | Effect Size | Lower CI | Upper CI | P Value | I Square |
|---|---|---|---|---|---|
| Angellotti et al., 2018 | -0.2476 | -0.5379 | 0.0427 | 0.2744 | 94.7339 |
| Barale et al., 2020 | -0.2270 | -0.5079 | 0.0539 | 0.3005 | 94.7347 |
| Barchetta et al., 2016 | -0.2697 | -0.5500 | 0.0107 | 0.2176 | 94.6193 |
| Breslavsky et al., 2013 | -0.2334 | -0.5163 | 0.0495 | 0.2903 | 94.7349 |
| Dadrass et al., 2019 | -0.0414 | -0.3026 | 0.2198 | 0.8390 | 94.1002 |
| Dalan et al., 2016 | -0.2617 | -0.5440 | 0.0206 | 0.2349 | 94.6666 |
| Gagnon et al., 2014 | -0.3143 | -0.5753 | -0.0532 | 0.1229 | 93.7747 |
| Hajj et al., 2020 | -0.2143 | -0.4998 | 0.0713 | 0.3362 | 94.6749 |
| Hoseini et al.., 2022 | -0.0597 | -0.3215 | 0.2021 | 0.7701 | 94.1810 |
| Hu et al., 2022 | -0.2352 | -0.5324 | 0.0621 | 0.3106 | 94.6112 |
| Imanparast et al., 2020 | 0.0277 | -0.2213 | 0.2767 | 0.8866 | 93.3761 |
| Kampmann et al., 2014 | -0.2230 | -0.5017 | 0.0557 | 0.3051 | 94.7348 |
| Mirzavandi et al., 2020 | -0.2940 | -0.5680 | -0.0200 | 0.1691 | 94.4074 |
| Muñoz-Aguirre et al., 2014 | -0.2342 | -0.5228 | 0.0543 | 0.2982 | 94.7265 |
| Omidian et al., 2019 (a) | -0.2250 | -0.5097 | 0.0596 | 0.3109 | 94.7249 |
| Punthakee et al., 2012 | -0.3191 | -0.6638 | 0.0256 | 0.2354 | 94.7280 |
| Ryu et al., 2014 | -0.2442 | -0.5284 | 0.0400 | 0.2708 | 94.7282 |
| Sadiya et al., 2015 | -0.2836 | -0.5604 | -0.0069 | 0.1891 | 94.3888 |
| Tabesh et al., 2014 | -0.0502 | -0.3080 | 0.2075 | 0.8028 | 93.6678 |
| Upreti et al.,2018 | -0.2923 | -0.5662 | -0.0184 | 0.1714 | 94.3644 |
| Wenclewska et al., 2019 | -0.2496 | -0.5301 | 0.0309 | 0.2540 | 94.7147 |
| Witham et al., 2010 | -0.1826 | -0.4617 | 0.0965 | 0.4017 | 94.6334 |

**Table S9: Sensitivity analysis for HDL using leave-one analysis**

| Deleted Study | Effect Size | Lower CI | Upper CI | P Value | I Square |
|---|---|---|---|---|---|
| Angellotti et al., 2018 | 0.5437 | 0.2215 | 0.8660 | 0.0306 | 95.3078 |
| Barale et al., 2020 | 0.5132 | 0.2032 | 0.8233 | 0.0339 | 95.3119 |

| Deleted Study | Effect Size | Lower CI | Upper CI | P Value | I Square |
|---|---|---|---|---|---|
| Barchetta et al., 2016 | 0.5847 | 0.2753 | 0.8940 | 0.0154 | 95.1917 |
| Breslavsky et al., 2013 | 0.5450 | 0.2321 | 0.8579 | 0.0256 | 95.3204 |
| Dadrass et al., 2019 | 0.5175 | 0.2081 | 0.8269 | 0.0321 | 95.3191 |
| Dalan et al., 2016 | 0.5452 | 0.2305 | 0.8599 | 0.0264 | 95.3181 |
| Gagnon et al., 2014 | 0.5850 | 0.2747 | 0.8953 | 0.0157 | 95.1490 |
| Hajj et al., 2020 | 0.3638 | 0.0958 | 0.6317 | 0.0819 | 93.6070 |
| Hoseini et al.., 2022 | 0.6817 | 0.3917 | 0.9717 | 0.0026 | 94.8732 |
| Imanparast et al., 2020 | 0.5058 | 0.1944 | 0.8172 | 0.0374 | 95.2871 |
| Kampmann et al., 2014 | 0.5461 | 0.2382 | 0.8540 | 0.0230 | 95.3225 |
| Meng et al., 2020 | 0.5371 | 0.2151 | 0.8591 | 0.0325 | 95.3229 |
| Mirzavandi et al., 2020 | 0.5295 | 0.2160 | 0.8430 | 0.0304 | 95.3256 |
| Muñoz-Aguirre et al., 2014 | 0.5378 | 0.2171 | 0.8584 | 0.0316 | 95.3230 |
| Omidian et al., 2019 (a) | 0.5390 | 0.2234 | 0.8546 | 0.0286 | 95.3245 |
| Punthakee et al., 2012 | 0.5201 | 0.1450 | 0.8952 | 0.0756 | 95.0597 |
| Ryu et al., 2014 | 0.5391 | 0.2240 | 0.8542 | 0.0283 | 95.3246 |
| Sadiya et al., 2015 | 0.3275 | 0.0655 | 0.5895 | 0.1091 | 93.3804 |
| Tabesh et al., 2014 | 0.3863 | 0.1013 | 0.6713 | 0.0824 | 94.4296 |
| Upreti et al.,2018 | 0.4594 | 0.1563 | 0.7625 | 0.0521 | 95.0039 |

| Deleted Study | Effect Size | Lower CI | Upper CI | P Value | I Square |
|---|---|---|---|---|---|
| Wenclewska et al., 2019 | 0.4778 | 0.1710 | 0.7846 | 0.0459 | 95.2203 |

**Table S10: Sensitivity analysis for LDL using leave-one analysis**

| Deleted Study | Effect Size | Lower CI | Upper CI | P Value | I Square |
|---|---|---|---|---|---|
| Angellotti et al., 2018 | -0.0841 | -0.2978 | 0.1296 | 0.6142 | 90.1515 |
| Barale et al., 2020 | -0.0878 | -0.2939 | 0.1184 | 0.5854 | 90.1365 |
| Barchetta et al., 2016 | -0.1055 | -0.3122 | 0.1012 | 0.5130 | 89.9686 |
| Breslavsky et al., 2013 | -0.0980 | -0.3051 | 0.1090 | 0.5441 | 90.0681 |
| Dadrass et al., 2019 | -0.0481 | -0.2523 | 0.1561 | 0.7630 | 90.0126 |
| Dalan et al., 2016 | -0.0961 | -0.3045 | 0.1122 | 0.5542 | 90.0771 |
| Gagnon et al., 2014 | -0.1778 | -0.3521 | -0.0035 | 0.1910 | 85.6410 |
| Hajj et al., 2020 | 0.0084 | -0.1810 | 0.1978 | 0.9548 | 87.7085 |
| Hoseini et al.., 2022 | 0.0169 | -0.1750 | 0.2088 | 0.9103 | 88.8620 |
| Imanparast et al., 2020 | -0.0698 | -0.2775 | 0.1379 | 0.6666 | 90.1304 |
| Kampmann et al., 2014 | -0.0460 | -0.2490 | 0.1570 | 0.7715 | 90.0229 |
| Meng et al., 2020 | -0.1141 | -0.3213 | 0.0932 | 0.4806 | 89.5787 |
| Mirzavandi et al., 2020 | -0.0791 | -0.2874 | 0.1291 | 0.6263 | 90.1523 |
| Muñoz-Aguirre et al., 2014 | -0.0784 | -0.2908 | 0.1341 | 0.6364 | 90.1461 |
| Omidian et al., 2019 (a) | -0.0777 | -0.2873 | 0.1318 | 0.6345 | 90.1500 |

| Deleted Study | Effect Size | Lower CI | Upper CI | P Value | I Square |
|---|---|---|---|---|---|
| Punthakee et al., 2012 | -0.0994 | -0.3502 | 0.1514 | 0.6115 | 90.1177 |
| Ryu et al., 2014 | -0.1005 | -0.3084 | 0.1074 | 0.5356 | 90.0279 |
| Sadiya et al., 2015 | -0.0623 | -0.2719 | 0.1472 | 0.7030 | 90.0300 |
| Tabesh et al., 2014 | 0.0342 | -0.1499 | 0.2183 | 0.8117 | 87.2953 |
| Upreti et al.,2018 | -0.0571 | -0.2647 | 0.1505 | 0.7244 | 90.0086 |
| Wenclewska et al., 2019 | -0.0944 | -0.3004 | 0.1115 | 0.5568 | 90.1041 |

**Publication bias.**

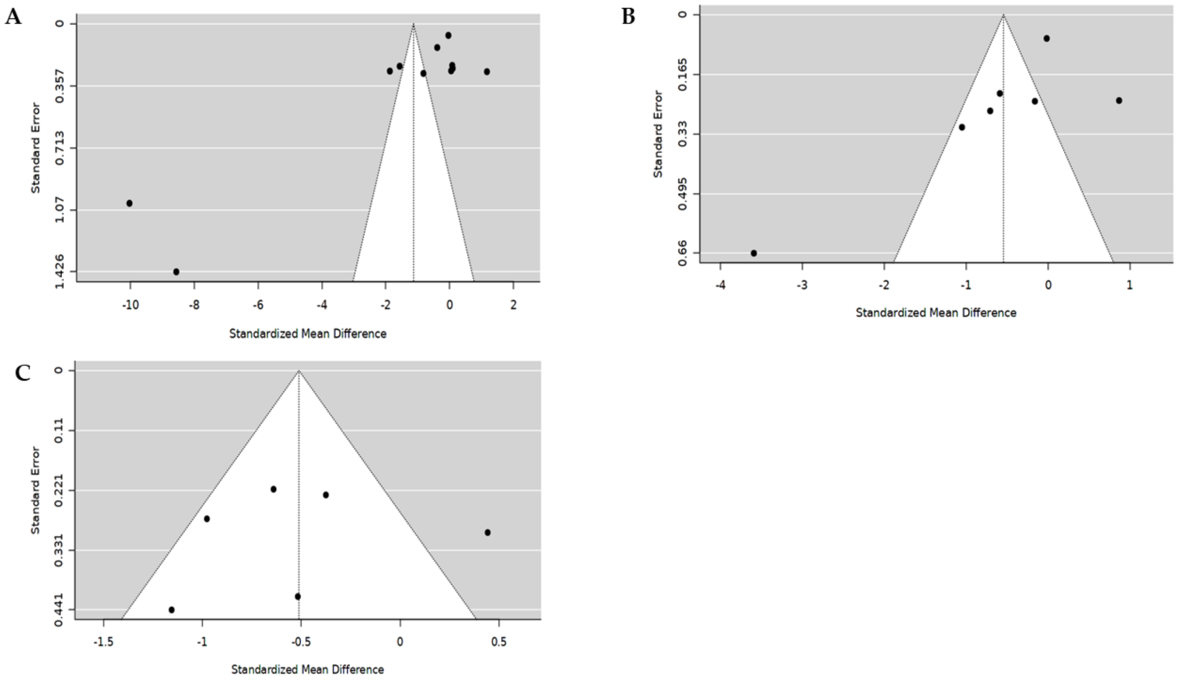


Figure S1: Funnel plots showing bias on inflammatory markers. A-High sensitivty -C-reactive protein (hs-CRP), B-Interleukin-6 (IL-6), C-Tumor necrosis factor alpha (TNF-$\alpha$).

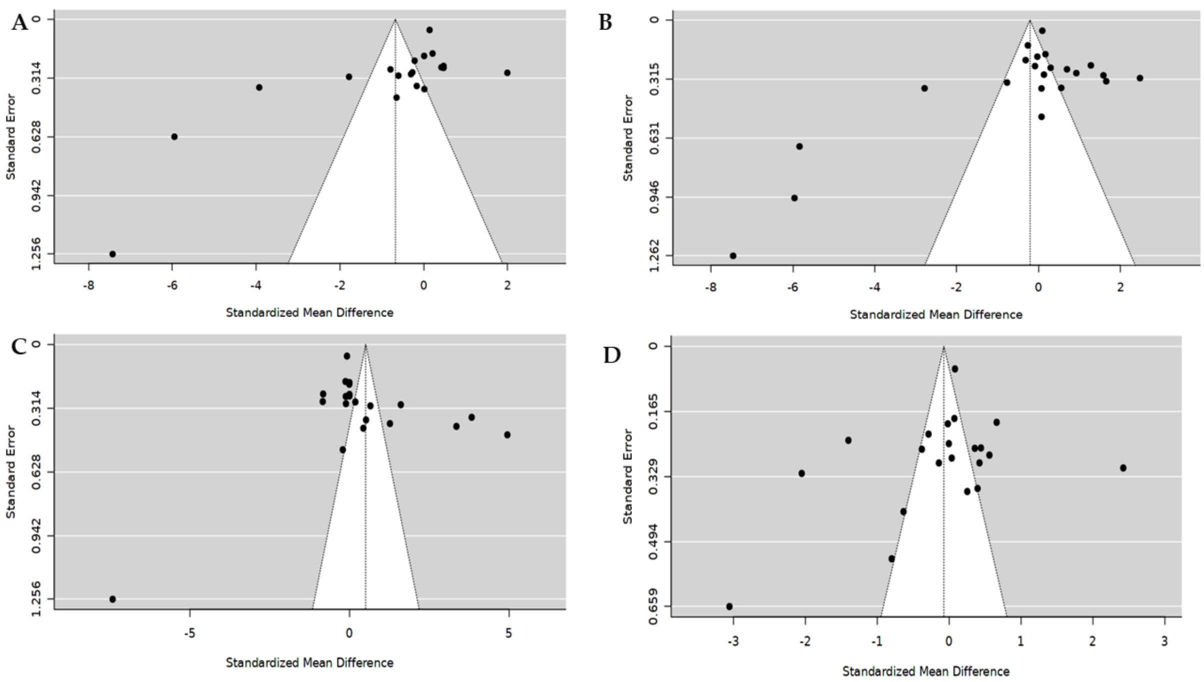


**Figure S2:** Funnel plots showing bias on lipid profiles. A-Total cholesterol, B-Triglyceride, C-High-density lipoprotein, D-Low-density lipoprotein

**Table S11:** Summary of findings table to evaluate certainty of evidence

**Vitamin D compared to placebo initial for [type 2 diabetes]**

**Patient or population:** [Adults with type 2 diabetes]

**Setting: Randomized controlled trials**

**Intervention:** Vitamin D

**Comparison:** placebo

| Outcomes | № of participants (studies) Follow-up | Certainty of the evidence (GRADE) | Relative effect (95% CI) | Anticipated absolute effects | |
|---|---|---|---|---|---|
| | | | | Risk with placebo intital | Risk difference with Vitamin D |
| Tumor necrosis-factor-alpha (TNF-α) | 321 (6 RCTs) | ⊕⊕⊕⊕ High[a,b] | - | - | SMD **0.51 SD lower** (0.93 lower to 0.09 lower) |
| Interleukin-6 (IL-6) | 1301 (7 RCTs) | ⊕⊕⊕⊕ High[b,c] | - | - | SMD **0.52 lower** (1.05 lower to 0 ) |
| High sensitivity-C-reactive protein (hs-CRP) | 1676 (11 RCTs) | ⊕⊕⊕◯ Moderate[c,d] | - | - | SMD 1.06 **lower** (1.67 lower to 0.45 lower) |
| Triglyceride (TG) | 2300 (19 RCTs) | ⊕⊕⊕⊕ High[d,e] | - | - | SMD **0.65 SD lower** (1.11 lower to 0.18 lower) |

**Vitamin D compared to placebo initial for [type 2 diabetes]**

**Patient or population:** [Adults with type 2 diabetes]

**Setting: Randomized controlled trials**

**Intervention:** Vitamin D

**Comparison:** placebo

| Outcomes | № of participants (studies) Follow-up | Certainty of the evidence (GRADE) | Relative effect (95% CI) | Anticipated absolute effects | |
|---|---|---|---|---|---|
| | | | | Risk with placebo intital | Risk difference with Vitamin D |
| Total Cholesterol (TC) | 2575 (22 RCTs) | ⊕⊕⊕⊕ High[a,d] | - | - | SMD **0.16 lower** (0.57 lower to 0.24 higher) |
| High-density lipoprotein (HDL) | 2430 (21 RCTs) | ⊕⊕⊕◯ Moderate[c,d] | - | - | SMD **0.53 higher** (0.08 higher to 0.98 higher) |
| Low-density-lipoprotein (LDL) | 2430 (21 RCTs) | ⊕⊕◯◯ Low[c,d] | - | - | SMD **0.06 lower** (0.37 lower to 0.24 higher) |

***The risk in the intervention group** (and its 95% confidence interval) is based on the assumed risk in the comparison group and the **relative effect** of the intervention (and its 95% CI).

**CI:** confidence interval; **SMD:** standardised mean difference

| Vitamin D compared to placebo initial for [type 2 diabetes] | | | | | |
|---|---|---|---|---|---|
| **Patient or population:** [Adults with type 2 diabetes]<br>**Setting: Randomized controlled trials**<br>**Intervention:** Vitamin D<br>**Comparison:** placebo | | | | | |
| **Outcomes** | **№ of participants (studies) Follow-up** | **Certainty of the evidence (GRADE)** | **Relative effect (95% CI)** | **Anticipated absolute effects** | |
| | | | | **Risk with placebo intital** | **Risk difference with Vitamin D** |
| **GRADE Working Group grades of evidence**<br>**High certainty:** we are very confident that the true effect lies close to that of the estimate of the effect.<br>**Moderate certainty:** we are moderately confident in the effect estimate: the true effect is likely to be close to the estimate of the effect, but there is a possibility that it is substantially different.<br>**Low certainty:** our confidence in the effect estimate is limited: the true effect may be substantially different from the estimate of the effect.<br>**Very low certainty:** we have very little confidence in the effect estimate: the true effect is likely to be substantially different from the estimate of effect. | | | | | |

**Explanations**

a. These trials showed a, moderate heterogeneity, which was reduced following subgroup analysis according to the duration of the intervention.

b. The funnel plot revealed a symmetrical shape, which suggests the absence of publication bias.

c. High statistical heterogeneity, which was not reduced following subgroup analysis.

d. The funnel plot asymmetrical shape suggests the presence of bias.

e. Studies showed a statistical heterogeneity however, this was reduced following subgroup analysis according to duration, especially when vitamin D was administered over a log period.

## References


1. Sadiya, A.; Ahmed, S.M.; Carlsson, M.; Tesfa, Y.; George, M.; Ali, S.H.; Siddieg, H.H.; Abusnana, S. Vitamin D Supplementation in Obese Type 2 Diabetes Subjects in Ajman, UAE: A Randomized Controlled Double-Blinded Clinical Trial. *Eur. J. Clin. Nutr.* **2015**, *69*, 707–711. https://doi.org/10.1038/ejcn.2014.251.
2. Neyestani, T.R.; Nikooyeh, B.; Alavi-Majd, H.; Shariatzadeh, N.; Kalayi, A.; Tayebinejad, N.; Heravifard, S.; Salekzamani, S.; Zahedirad, M. Improvement of Vitamin D Status via Daily Intake of Fortified Yogurt Drink Either with or without Extra Calcium Ameliorates Systemic Inflammatory Biomarkers, Including Adipokines, in the Subjects with Type 2 Diabetes. *J. Clin. Endocrinol. Metab.* **2012**, *97*, 2005–2011. https://doi.org/10.1210/jc.2011-3465.
3. Akbarzadeh, M.; Eftekhari, M.H.; Dabbaghmanesh, M.H.; Hasanzadeh, J.; Bakhshayeshkaram, M. Serum IL-18 and HsCRP Correlate with Insulin Resistance without Effect of Calcitriol Treatment on Type 2 Diabetes. *Iran. J. Immunol.* **2013**, *10*, 167–176.
4. Tabesh, M.; Azadbakht, L.; Faghihimani, E.; Tabesh, M.; Esmaillzadeh, A. Effects of Calcium–Vitamin D Co-Supplementation on Metabolic Profiles in Vitamin D Insufficient People with Type 2 Diabetes: A Randomised Controlled Clinical Trial. *Diabetologia* **2014**, *57*, 2038–2047. https://doi.org/10.1007/s00125-014-3313-x.
5. Fazelian, S.; Paknahad, Z.; Khajehali, L.; Kheiri, S.; Amani, R. The Effects of Supplementation with Vitamin D on Inflammatory Biomarkers, Omentin, and Vaspin in Women with Type 2 Diabetes: A Randomized Double-Blind Placebo-Controlled Clinical Trial. *J. Food Biochem.* **2018**, *42*, e12631. https://doi.org/10.1111/jfbc.12631.
6. Omidian, M.; Mahmoudi, M.; Abshirini, M.; Eshraghian, M.R.; Javanbakht, M.H.; Zarei, M.; Hasani, H.; Djalali, M. Effects of Vitamin D Supplementation on Depressive Symptoms in Type 2 Diabetes Mellitus Patients: Randomized Placebo-Controlled Double-Blind Clinical Trial. *Diabetes Metab. Syndr. Clin. Res. Rev.* **2019**, *13*, 2375–2380. https://doi.org/10.1016/j.dsx.2019.06.011.
7. Omidian, M.; Mahmoudi, M.; Javanbakht, M.H.; Eshraghian, M.R.; Abshirini, M.; Daneshzad, E.; Hasani, H.; Alvandi, E.; Djalali, M. Effects of Vitamin D Supplementation on Circulatory YKL-40 and MCP-1 Biomarkers Associated with Vascular Diabetic Complications: A Randomized, Placebo-Controlled, Double-Blind Clinical Trial. *Diabetes Metab. Syndr. Clin. Res. Rev.* **2019**, *13*, 2873–2877. https://doi.org/10.1016/j.dsx.2019.07.047.
8. Dadrass, A.; Mohamadzadeh Salamat, K.; Hamidi, K.; Azizbeigi, K. Anti-Inflammatory Effects of Vitamin D and Resistance Training in Men with Type 2 Diabetes Mellitus and Vitamin D Deficiency: A Randomized, Double-Blinded, Placebo-Controlled Clinical Trial. *J. Diabetes Metab. Disord.* **2019**, *18*, 323–331. https://doi.org/10.1007/s40200-019-00416-z.
9. Mirzavandi, F.; Talenezhad, N.; Razmpoosh, E.; Nadjarzadeh, A.; Mozaffari-Khosravi, H. The Effect of Intramuscular Megadose of Vitamin D Injections on E-Selectin, CRP and Biochemical Parameters in Vitamin D-Deficient Patients with Type-2 Diabetes Mellitus: A Randomized Controlled Trial. *Complement. Ther. Med.* **2020**, *49*, 102346. https://doi.org/10.1016/j.ctim.2020.102346.
10. Imanparast, F.; Javaheri, J.; Kamankesh, F.; Rafiei, F.; Salehi, A.; Mollaaliakbari, Z.; Rezaei, F.; Rahimi, A.; Abbasi, E. The Effects of Chromium and Vitamin D3 Co-Supplementation on Insulin Resistance and Tumor Necrosis Factor-Alpha in Type 2 Diabetes: A Randomized Placebo-Controlled Trial. *Appl. Physiol. Nutr. Metab.* **2019**, *45*, 471–477. https://doi.org/10.1139/apnm-2019-0113.
11. Hoseini, R.; Rahim, H.A.; Ahmed, J.K. Decreased Inflammatory Gene Expression Accompanies the Improvement of Liver Enzyme and Lipid Profile Following Aerobic Training and Vitamin D Supplementation in T2DM Patients. *BMC Endocr. Disord.* **2022**, *22*, 245. https://doi.org/10.1186/s12902-022-01152-x.
12. Limonte, C.P.; Zelnick, L.R.; Ruzinski, J.; Hoofnagle, A.N.; Thadhani, R.; Melamed, M.L.; Lee, I.M.; Buring, J.E.; Sesso, H.D.; Manson, J.A.E.; et al. Effects of Long-Term Vitamin D and n-3 Fatty Acid Supplementation on Inflammatory and Cardiac Biomarkers in Patients with Type 2 Diabetes: Secondary Analyses from a Randomised Controlled Trial. *Diabetologia* **2021**, *64*, 437–447. https://doi.org/10.1007/s00125-020-05300-7.
13. Meng, H.; Matthan, N.R.; Angellotti, E.; Pittas, A.G.; Lichtenstein, A.H. Exploring the Effect of Vitamin D3 Supplementation on Surrogate Biomarkers of Cholesterol Absorption and Endogenous Synthesis in Patients with Type 2 Diabetes-Randomized Controlled Trial. *Am. J. Clin. Nutr.* **2020**, *112*, 538–547. https://doi.org/10.1093/ajcn/nqaa149.
14. Angellotti, E.; D'Alessio, D.; Dawson-Hughes, B.; Chu, Y.; Nelson, J.; Hu, P.; Cohen, R.M.; Pittas, A.G. Effect of Vitamin D Supplementation on Cardiovascular Risk in Type 2 Diabetes. *Clin. Nutr.* **2019**, *38*, 2449–2453. https://doi.org/10.1016/j.clnu.2018.10.003.
15. Witham, M.D.; Dove, F.J.; Dryburgh, M.; Sugden, J.A.; Morris, A.D.; Struthers, A.D. The Effect of Different Doses of Vitamin D3 on Markers of Vascular Health in Patients with Type 2 Diabetes: A Randomised Controlled Trial. *Diabetologia* **2010**, *53*, 2112–2119. https://doi.org/10.1007/s00125-010-1838-1.
16. Punthakee, Z.; Bosch, J.; Dagenais, G.; Diaz, R.; Holman, R.; Probstfield, J.L.; Ramachandran, A.; Riddle, M.C.; Rydén, L.E.; Zinman, B.; et al. Design, History and Results of the Thiazolidinedione Intervention with Vitamin D Evaluation (TIDE) Randomised Controlled Trial. *Diabetologia* **2012**, *55*, 36–45. https://doi.org/10.1007/s00125-011-2357-4.

17. Barchetta, I.; Del Ben, M.; Angelico, F.; Di Martino, M.; Fraioli, A.; La Torre, G.; Saulle, R.; Perri, L.; Morini, S.; Tiberti, C.; et al. No Effects of Oral Vitamin D Supplementation on Non-Alcoholic Fatty Liver Disease in Patients with Type 2 Diabetes: A Randomized, Double-Blind, Placebo-Controlled Trial. *BMC Med.* **2016**, *14*, 92. https://doi.org/10.1186/s12916-016-0638-y.
18. Barale, M.; Rossetto Giaccherino, R.; Ghigo, E.; Procopio, M. Effect of 1-Year Oral Cholecalciferol on a Metabolic Profile and Blood Pressure in Poor-Controlled Type 2 Diabetes Mellitus: An Open-Label Randomized Controlled Pilot Study. *J. Endocrinol. Investig.* **2021**, *44*, 791–802. https://doi.org/10.1007/s40618-020-01373-8.
19. Maggi, S.; Siviero, P.; Brocco, E.; Albertin, M.; Romanato, G.; Crepaldi, G. Vitamin D Deficiency, Serum Leptin and Osteoprotegerin Levels in Older Diabetic Patients: An Input to New Research Avenues. *Acta Diabetol.* **2014**, *51*, 461–469. https://doi.org/10.1007/s00592-013-0540-4.
20. Gagnon, C.; Daly, R.M.; Carpentier, A.; Lu, Z.X.; Shore-Lorenti, C.; Sikaris, K.; Jean, S.; Ebeling, P.R. Effects of Combined Calcium and Vitamin D Supplementation on Insulin Secretion, Insulin Sensitivity and β-Cell Function in Multi-Ethnic Vitamin D-Deficient Adults at Risk for Type 2 Diabetes: A Pilot Randomized, Placebo-Controlled Trial. *PLoS ONE* **2014**, *9*, e109607. https://doi.org/10.1371/journal.pone.0109607.
21. Hu, Z.; Zhi, X.; Li, J.; Li, B.; Wang, J.; Zhu, J.; Zhang, Z. Effects of Long-Term Vitamin D Supplementation on Metabolic Profile in Middle-Aged and Elderly Patients with Type 2 Diabetes. *J. Steroid Biochem. Mol. Biol.* **2022**, *225*, 106198. https://doi.org/10.1016/j.jsbmb.2022.106198.
22. Kampmann, U.; Mosekilde, L.; Juhl, C.; Moller, N.; Christensen, B.; Rejnmark, L.; Wamberg, L.; Orskov, L. Effects of 12 Weeks High Dose Vitamin D3 Treatment on Insulin Sensitivity, Beta Cell Function, and Metabolic Markers in Patients with Type 2 Diabetes and Vitamin D Insufficiency—A Double-Blind, Randomized, Placebo-Controlled Trial. *Metabolism* **2014**, *63*, 1115–1124. https://doi.org/10.1016/j.metabol.2014.06.008.
23. Upreti, V.; Maitri, V.; Dhull, P.; Handa, A.; Prakash, M.S.; Behl, A. Effect of Oral Vitamin D Supplementation on Glycemic Control in Patients with Type 2 Diabetes Mellitus with Coexisting Hypovitaminosis D: A Parellel Group Placebo Controlled Randomized Controlled Pilot Study. *Diabetes Metab. Syndr. Clin. Res. Rev.* **2018**, *12*, 509–512. https://doi.org/10.1016/j.dsx.2018.03.008.
24. Breslavsky, A.; Frand, J.; Matas, Z.; Boaz, M.; Barnea, Z.; Shargorodsky, M. Effect of High Doses of Vitamin D on Arterial Properties, Adiponectin, Leptin and Glucose Homeostasis in Type 2 Diabetic Patients. *Clin. Nutr.* **2013**, *32*, 970–975. https://doi.org/10.1016/j.clnu.2013.01.020.
25. Ryu, O.H.; Chung, W.; Lee, S.; Hong, K.S.; Choi, M.G.; Yoo, H.J. The Effect of High-Dose Vitamin D Supplementation on Insulin Resistance and Arterial Stiffness in Patients with Type 2 Diabetes. *Korean J. Intern. Med.* **2014**, *29*, 620–629. https://doi.org/10.3904/kjim.2014.29.5.620.
26. Muñoz-Aguirre, P.; Flores, M.; Macias, N.; Quezada, A.D.; Denova-Gutiérrez, E.; Salmerón, J. The Effect of Vitamin D Supplementation on Serum Lipids in Postmenopausal Women with Diabetes: A Randomized Controlled Trial. *Clin. Nutr.* **2015**, *34*, 799–804. https://doi.org/10.1016/j.clnu.2014.10.002.
27. Dalan, R.; Liew, H.; Assam, P.N.; Chan, E.S.Y.; Siddiqui, F.J.; Tan, A.W.K.; Chew, D.E.K.; Boehm, B.O.; Leow, M.K.S. A Randomised Controlled Trial Evaluating the Impact of Targeted Vitamin D Supplementation on Endothelial Function in Type 2 Diabetes Mellitus: The DIMENSION Trial. *Diab Vasc. Dis. Res.* **2016**, *13*, 192–200. https://doi.org/10.1177/1479164115621667.
28. Jehle, S.; Lardi, A.; Felix, B.; Hulter, H.N.; Stettler, C.; Krapf, R. Effect of Large Doses of Parenteral Vitamin D on Glycaemic Control and Calcium/Phosphate Metabolism in Patients with Stable Type 2 Diabetes Mellitus: A Randomised, Placebo-Controlled, Prospective Pilot Study. *Swiss Med. Wkly.* **2014**, *144*, w13942. https://doi.org/10.4414/smw.2014.13942.
29. El Hajj, C.; Walrand, S.; Helou, M.; Yammine, K. Effect of Vitamin D Supplementation on Inflammatory Markers in Non-Obese Lebanese Patients with Type 2 Diabetes: A Randomized Controlled Trial. *Nutrients* **2020**, *12*, 2033. https://doi.org/10.3390/nu12072033.
30. Wenclewska, S.; Szymczak-Pajor, I.; Drzewoski, J.; Bunk, M.; Śliwińska, A. Vitamin d Supplementation Reduces Both Oxidative Dna Damage and Insulin Resistance in the Elderly with Metabolic Disorders. *Int. J. Mol. Sci.* **2019**, *20*, 28911. https://doi.org/10.3390/ijms20122891.
